# A scoping review on using real-world data to evaluate the effectiveness of mHealth applications

**DOI:** 10.1038/s41746-026-02562-0

**Published:** 2026-04-08

**Authors:** Sara Gehder, Stefanie Brückner, Stephen Gilbert, Moritz Goeldner

**Affiliations:** 1 Data-Driven Innovation, Hamburg University of Technology, Hamburg, Germany; 2 Else Kröner Fresenius Center for Digital Health, TUD Dresden University of Technology, Dresden, Germany; 3 Faculty of Business and Economics, TUD Dresden University of Technology, Dresden, Germany

**Keywords:** Business and industry, Health care, Medical research

## Abstract

Mobile health (mHealth) applications are increasingly integral to healthcare delivery, yet traditional randomized trials face practical challenges in evaluating these dynamic tools. Real-world data (RWD), collected during routine app use, offers a complementary pathway to real-world evidence (RWE) that may reflect how mHealth applications are used in everyday settings. We conducted a scoping review to map how naturally emerging RWD are currently used in peer-reviewed studies to evaluate patient-facing mHealth applications. We systematically searched PubMed, Scopus, and Web of Science (January 2007–November 2024) and extracted data on application type, RWD characteristics and study design aspects. Study-level, design-centred evidence levels for RWD-based effectiveness evaluations were assigned using a combined Oxford Centre for Evidence-Based Medicine and FDA RWE framework. Seventy-two studies evaluating 61 unique mHealth applications were included. Most studies focused on mental health or metabolic conditions and relied predominantly on data actively reported by users, often via in-app surveys, with comparatively limited use of device-generated data or integration with system-generated data such as clinical or claims data. Single-group pre–post approaches were most frequently observed, while only a minority employed comparative observational, quasi-experimental, or randomized designs. These findings illustrate current patterns in the use of RWD for mHealth evaluation and highlight both opportunities and constraints in longitudinal and comparative assessments of mHealth applications in real-world contexts.

## Introduction

The integration of mobile and wireless technologies into healthcare (mHealth) is transforming health service delivery globally, particularly in resource-limited and remote settings, contributing to Sustainable Development Goal 3 on universal health and well-being^1,2^. Central to this transformation are mobile health applications, now widely adopted, with over 337,000 health apps listed in Apple App Store and Google Play Store^3^, and an estimated 200 new digital health apps released daily across app stores^3^. Patients value their accessibility and personalization^4^, and many apps now support chronic disease self-management and behavior change.

To support the safe and effective use of digital health technologies, regulatory and reimbursement pathways, such as Germany’s DiGA pathway^5^ (app on prescription) and the UK’s NICE Evidence Standards Framework^6^, alongside similar initiatives internationally^7–10^, have introduced structured evidence expectations^11,12^. In parallel, real-world data (RWD) is playing an increasingly important role in regulatory decision-making, market access, and post-market performance monitoring for mHealth interventions^13–16^.

Robust evidence is critical for enabling these pathways. Clinicians and payers alike emphasize the need for stronger evidence bases to inform decisions about the use of mobile health applications in practice^17^. However, evaluating the effectiveness of software-based health interventions presents methodological challenges. Traditional randomized controlled trials (RCTs), while considered the gold standard for establishing causal effectiveness of interventions, face practical challenges for digital tools due to rapid product iteration cycles, personalized delivery and the integration into care pathways^18–21^. Another methodological challenge is the proper blinding of patients and providers. Digital placebos, so-called sham apps, that mimic the digital intervention without the active therapeutic component, have been introduced but require further investigation for widespread application^22^. RWD, defined as routinely collected, longitudinal health data from everyday clinical or user interactions^23^, is increasingly recognized as an important complementary data source to generate real-world evidence (RWE) and regulatory guidance for its implementation is evolving^24,25^.

However, the quality and validity of RWE, defined as “the clinical evidence regarding the usage and potential benefits or risks of a medical product derived from analysis of RWD”^23^, depends on the quality of the RWD source, how it is generated and how it is evaluated to yield meaningful insights into the performance of mHealth applications. Key considerations include whether data are captured as part of routine product use or introduced solely for research purposes, and whether collection is passive (e.g., through sensors or usage logs) or active (e.g., user-entered symptom tracking). Structured, unobtrusive in-app data capture is generally considered to better reflect real-world use compared to study-specific instruments administered externally, which may introduce bias or alter user behavior^26–28^. Moreover, the strength of the resulting RWE is determined by study design, including the presence of comparators and the use of causal inference methods, in alignment with guidance from frameworks such as the Oxford Centre for Evidence-Based Medicine (OCEBM)^29^ and the FDA’s RWE guidance^23^.

Despite the potential of mHealth applications to generate valuable RWD, it remains unclear how extensively such data are used in the peer-reviewed literature to evaluate real-world effectiveness. In some cases, apps may lack the infrastructure to support data generation; in others, available data may remain underused due to technical, methodological, or regulatory barriers. To better understand this landscape, we examined published studies as a proxy for current practice. Published evaluations provide insight into how RWD are currently used and reported in the peer-reviewed literature and therefore serve as a practical lens for understanding the current state of evidence generation. At the same time, existing evaluations span highly heterogeneous application types, clinical domains, data sources, outcome constructs, and study designs, and no standardized framework yet exists to structure RWD-based evaluation of mHealth interventions. Against this backdrop, a scoping review allows a structured mapping of how RWD are currently being used across these dimensions.

To this end, we conducted a scoping review to systematically map the landscape of published studies evaluating mHealth applications using RWD. Our analysis focused on patient-facing applications that collect “naturally emerging” RWD from routine app use, without the use of study-specific data collection instruments. We considered three categories of RWD: user input, device-generated, and system-generated data.

Our review addressed the following questions: To what extent is RWD generated through the routine use of mHealth applications used to (continuously) demonstrate their effectiveness in improving care, as reflected in peer-reviewed studies?

1. Which types of patient-focused mHealth applications currently incorporate naturally emerging RWD in studies for effectiveness evaluation?
2. What categories and types of naturally emerging RWD from routine app use are being reported in these studies?
3. Which study designs are used to assess the effectiveness of specific mHealth applications using naturally emerging RWD, and how do they align with regulatory and scientific recommendations?

By synthesizing the current state of scientific practice, this scoping review aims to identify emerging patterns, gaps, and methodological challenges in leveraging RWD to evaluate how effectiveness is assessed in mHealth. In doing so, we seek to inform future research, guide regulatory development, and support more targeted validation frameworks for digital health technologies.

## Results

### Screening results

**Fig. 1:**
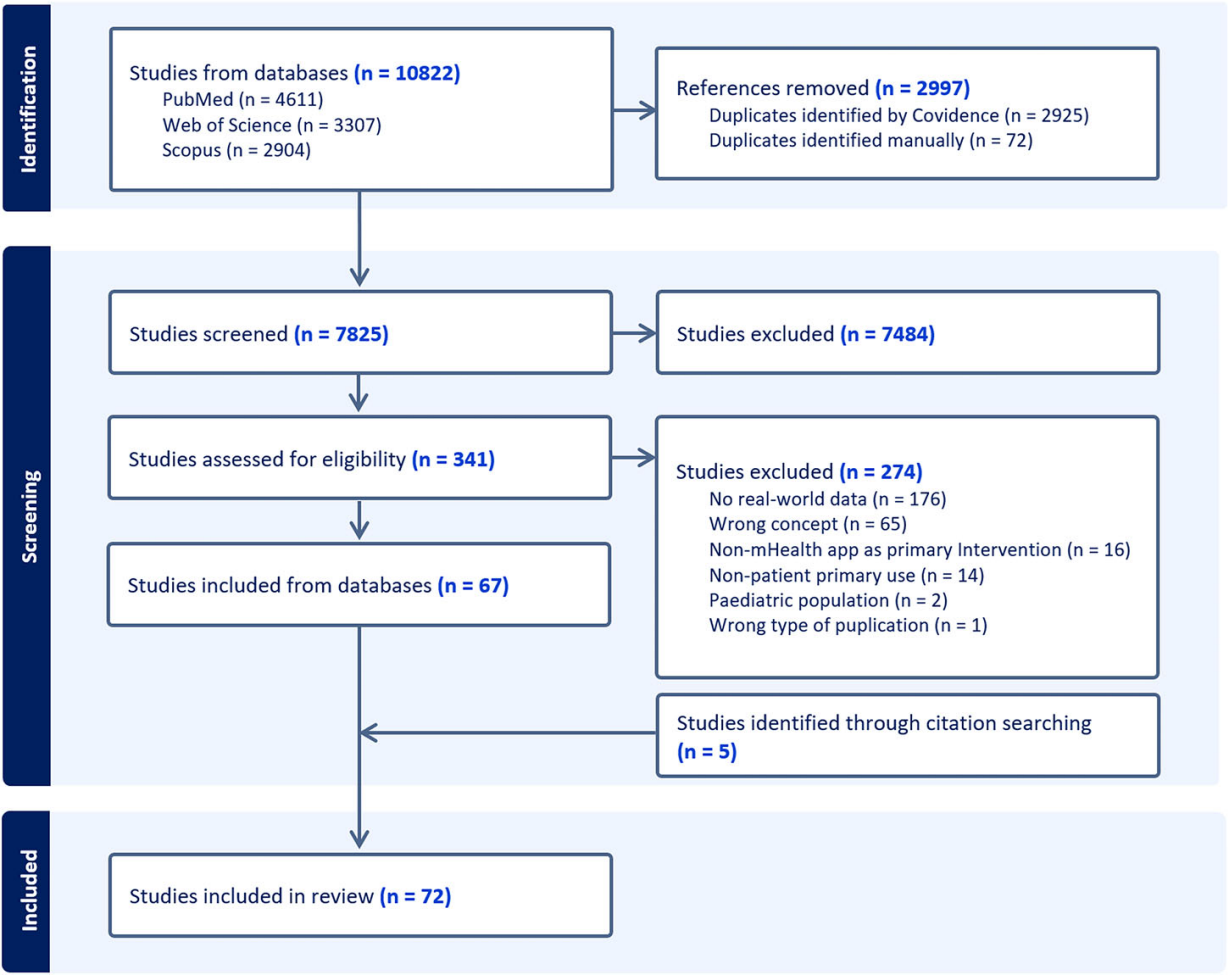
PRISMA Flow Diagram Illustrating the Study Selection Process.

Our search identified 10,822 records across three databases (Fig. 1). After removing duplicates, 7825 records underwent title and abstract screening, yielding 341 studies for full-text review. 67 studies met the eligibility criteria. Citation tracking of included studies identified 5 additional studies. In total, 72 studies were included.

### Characteristics of identified studies

The 72 included studies were published between 2016 and 2024, with an increase in yearly publications from 2021 onward. The studies spanned 16 countries, with the United States (*n* = 28) most represented, followed by Germany (*n* = 6), China (*n* = 5), the United Kingdom (*n* = 5), and France (*n* = 4). Seven studies were multinational, while the country of origin was unclear in three studies. Among the 72 included studies, 61 distinct mHealth applications were evaluated. Three applications were assessed in three studies each, and five additional applications appeared in two studies each. Full study-level characteristics with citations are provided in Supplementary Table 2.

### Characteristics of mHealth applications

To assess the medical device (MD) status of the 61 patient-facing mHealth applications included in the studies, we reviewed whether each app met the criteria for classification under the EU Medical Device Regulation (MDR)^30^ and the accompanying guidance MDCG 2021-24^31^. None of the included studies explicitly reported device classification. However, for 16 applications, regulatory status was confirmed via developer websites or the EUDAMED database. For the remaining tools, MD classification was inferred based on publicly available product descriptions of their intended medical use. This was combined with an assignment of their respective mHealth category, drawing on a hybrid framework adapted from Olla & Shimskey^32^ and the NICE Evidence Standards Framework^33^. Based on this assessment, 48 applications met the criteria for medical devices (Treatment, Diagnosis, guiding medical choices), while 13 fell into the general health and wellness category. While this does not constitute a formal regulatory designation, it provides a consistent overview of risk classification.

Twenty-six applications were designed to treat specific conditions, 16 to inform clinical management, and six to drive clinical decision-making. No application was classified under diagnostic use. Table 1 summarizes the number of applications and associated studies per potential regulatory status and mHealth category.

**Table 1:** Number of apps and studies across potential regulatory status and mHealth category

Potential Regulatory Status	mHealth Category	# of apps	# of studies
Potentially General Health & Wellness Tool		**13**	**17**
	Health & Care Diaries	3	3
	Promoting good health	10	14
Potentially Medical Device		**48**	**55**
	Inform Clinical Management	16	17
	Drive Clinical Management	6	8
	Treat Specific Condition	26	30
	Diagnose Specific Condition	0	0
**Total**		**61**	**72**

The 61 applications addressed a variety of clinical areas, with the most common being mental health (*n* = 19), endocrine and metabolic disorders (*n* = 18), cardiovascular conditions (*n* = 7), and musculoskeletal disorders (*n* = 6). Additional applications focused on reproductive health (*n* = 4), nervous system disorders (*n* = 3), and genitourinary conditions (*n* = 1). Three applications addressed less common or cross-cutting clinical areas not listed above and are detailed in Table 2, which also presents the mapping between medical specialties and mHealth categories.

**Table 2:** Number of apps and studies across medical specialty and mHealth category

Medical Specialty	mHealth Category	# of apps	# of studies
Mental Health		**19**	**24**
	Treat Specific Condition	13	16
	Promoting good health	4	6
	Inform Clinical Management	1	1
	Drive Clinical Management	1	1
Endocrine and Metabolic System		**18**	**21**
	Treat Specific Condition	2	2
	Promoting Good Health	5	7
	Inform Clinical Management	9	10
	Drive Clinical Management	1	1
	Health & Care Diaries	1	1
Cardiovascular System		**7**	**7**
	Treat Specific Condition	2	2
	Inform Clinical Management	4	4
	Drive Clinical Management	1	1
General Wellbeing		**3**	**3**
	Health & Care Diaries	2	2
	Promoting Good Health	1	1
Nervous System		**3**	**3**
	Treat Specific Condition	2	2
	Inform Clinical Management	1	1
Musculoskeletal System	Treat Specific Condition	**6**	**7**
Reproductive health	Drive Clinical Management	**2**	**4**
Respiratory System	Drive Clinical Management	**1**	**1**
Cancer	Inform Clinical Management	**1**	**1**
Genitourinary System	Treat Specific Condition	**1**	**1**
**Total**		**61**	**72**

### Characteristics of real-world data

**Fig. 2:**
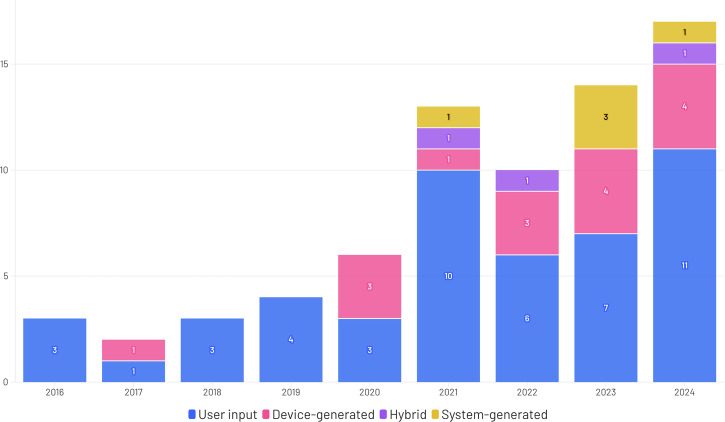
Number of Studies published per year per Data Category.

RWD characteristics were categorized according to a predefined taxonomy (Supplementary Note 2). App-collected user input data was the most frequently used category of RWD, appearing in 71% (51/72) of studies, followed by device-generated data in 26% (19/72) and system-generated data in 7% (5/72) (Table 3). Because three studies used more than one RWD category (e.g., user input combined with device-generated data), these proportions sum to more than 100%. These three studies are further labeled as hybrid. Fewer studies used system-generated data (7%, 5/72). As shown in Fig. 2, the first publications found using RWD focused mainly on user input data. Over the next few years, device-generated data or a combination of both will be increasingly used.

**Table 3:** Number of studies across data categories and data types. Studies may contribute data to more than one category or type; counts are therefore not mutually exclusive

RWD Categories	RWD Types	# studies
User Input Data		**51**
	In-App-Survey	35
	Vital Stats	12
	Event tags	4
Device-Generated Data		**19**
	Connected Device	15
	Activity Data	2
	Task Performance	1
	App Engagement	1
System-Generated Data		**5**
	Procedures (EHR)	1
	Admission/Discharge and Progress Reports (EHR)	1
	Vital Stats (EHR)	1
	Medication Use (claims data)	1
	Medication Use (claims), Admission/Discharge and Progress Reports (claims)	1

Among the studies using user input data, either alone (*n* = 48) or in combination with device-generated data (*n* = 3), the total number was 51. The most common data type in these studies was in-app surveys (68%, 35/51), capturing symptoms, disease severity, and pain scores. Within this group of studies, 63% (22/35) used validated instruments, such as Patient Health Questionnaire-9 (PHQ-9) or Insomnia Severity Index (ISI), while 37% (13/35) used surveys that were not described as established, previously validated Patient Reported Outcome Measures (PROMs) in the respective publications. Other studies collecting vital stats (24%, 12/51), such as body weight or blood pressure measurements, were also frequent, while event tags (8%, 4/51), including entries like smoking or unprotected sexual activity, were less common.

In total, 19 studies used device-generated data - 16 exclusively, and three in combination with user input data. The most frequently used data type in this group was connected devices (79%, 15/19), such as Bluetooth-enabled scales, Continuous Glucose Monitoring (CGM) devices, or activity trackers. User device-generated activity data (11%, 2/19) included passively recorded step counts, task performance data (5%, 1/19) assessed cognitive or motor function, and app engagement metrics (5%, 1/19) tracked user interaction frequency.

Studies using system-generated data equally relied on admission/discharge reports (20%, 1/5), procedures (20%, 1/5), and vital statistics from electronic health records (20%, 1/5). Medication data from claims were used in 20% of studies (1/5), and one study (20%, 1/5) used both medication data and admission/discharge reports.

**Fig. 3:**
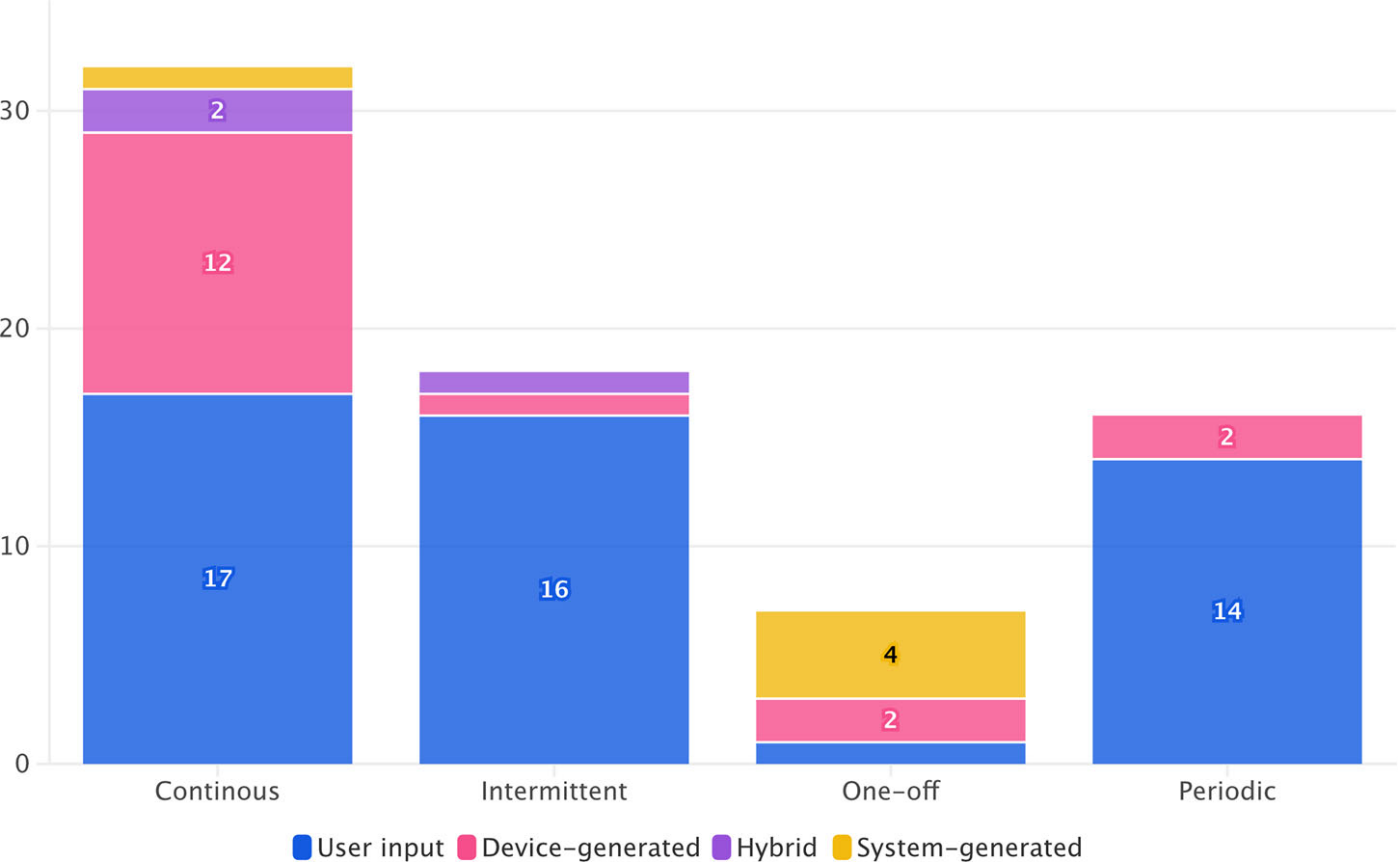
Distribution of Data Collection Frequency Across Data Categories in Identified Studies.

Among all studies, continuous data collection (44%, 32/72) was most common (Fig. 3). Continuous data collection in this review includes both truly continuous (e.g., passive sensor data) and continual (e.g., daily symptom trackers) modes with a high frequency of data collection (e.g., daily or at every app use). Intermittent collection (24%, 17/72) allowed participants to self-report data at chosen time points. Periodic collection (22%, 16/72) followed predefined intervals (other than daily), while one-off collection (10%, 7/72) involved a single data entry. In user input data studies (*n* = 51), continuous collection (38%, 19/51) and intermittent collection (33%, 17/51) were the most frequent, while periodic (27%, 14/51) and one-off (2%, 1/51) were less common. In device-generated data studies (*n* = 19), continuous collection (74%, 14/19) was predominant, while periodic (11%, 2/19) and one-off collection (11%, 2/19) were less frequent. In system-generated data studies, one-off data collection was the most frequent (80%, 4/5) while continuous data collection was less common (20%, 1/5).

### Use of RWD categories per medical specialty and mHealth application category

**Fig. 4:**
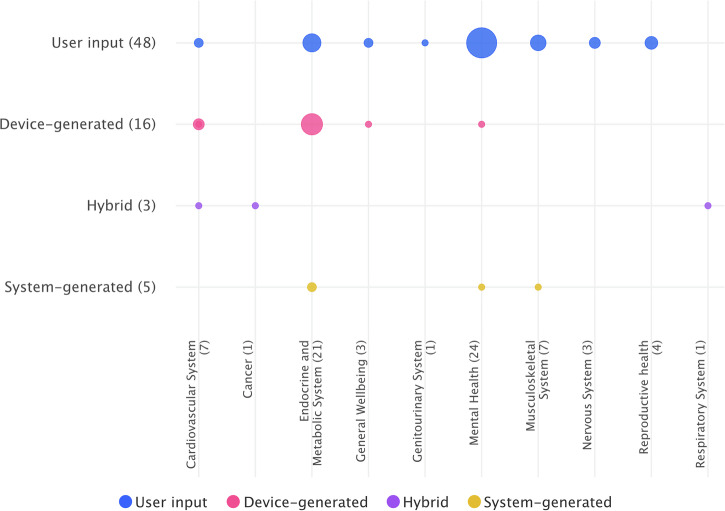
Distribution of real-world data categories across medical specialties in identified studies (Bubble sizes relate to the number of studies).

Among studies centered on mental health applications (*n* = 24), user input data dominated (92%, 22/24), while device-generated and system-generated data were each used in 4% (1/24). In studies on endocrine and metabolic applications (*n* = 21), device-generated data were most frequent (52%, 11/21), followed by user input (38%, 8/21) and system-generated data (10%, 2/21). Studies on musculoskeletal applications (*n* = 7) relied mostly on user input data (86%, 6/7), with one study (14%) using system-generated data. Among studies on cardiovascular applications (*n* = 7), device-generated data (43%, 3/7) and user input data (29%, 2/7) were most frequent, with combined user input and device-generated (14%, 1/7) and system-generated data (14%, 1/7) also used. Information for additional specialties and data types are summarized in Fig. 4.

For studies on applications treating specific conditions (*n* = 30), user input data was most used (83%, 25/30), followed by device-generated data (11%, 3/30), while system-generated data was used at 6% (2/30). For studies on applications informing clinical management (*n* = 17), device-generated data (59%, 10/17) was the most common RWD category, followed by user input data (24%, 4/17), while user input and device-generated data were combined in 12% (2/17), and system-generated data was used in 5% (1/17). Among studies on applications promoting good health (*n* = 14), user input data was dominant (79%, 11/14), with device-generated data used in 14% (2/14) and system-generated data in 6% (1/14). For studies on applications driving clinical management (*n* = 8), user input data was used in 75% (6/8), with user input and device-generated data combined in 13% (1/8), and system-generated data in 13% (1/8). In studies on health and care diary applications (*n* = 3), user input data was most common (67%, 2/3), while device-generated data was used in 33% (1/3).

### Study design characteristics and levels of evidence in effectiveness evaluation

Evidence levels were assigned using a hybrid classification based on the Oxford Centre for Evidence-Based Medicine (OCEBM) hierarchy^29^ and the FDA RWE framework^23^ (see Methods and Supplementary Note 3). Among the 72 included studies, the majority (57%, 41/72) were classified as level 4 evidence, using pre-post single-group designs with individual comparisons. Twenty-one studies (29%, 21/72) fell into level 3b, consisting of observational cohort studies that used intergroup comparisons without matched external comparators. Seven studies (10%, 7/72) were categorized as level 3a, employing quasi-experimental real-world evidence (RWE) designs with comparators. Finally, three studies (4%, 3/72) met criteria for level 2 evidence: one randomized controlled trial (RCT), one pragmatic randomized controlled trial (PCT), and one prospective cohort study with a concurrent control group.

Of the 72 studies, only 10 (14%, 10/72) included a real comparator such as a propensity score-matched or self-selected usual-care comparator. Two retrospective studies using system-generated data employed propensity score-matched usual-care comparators. In this group, the other three studies, using clinical data, were prospective and used either a randomized or self-selected usual-care comparator. Studies using device-generated data included one prospective study with a propensity score–matched comparator derived from clinical data and one retrospective study with a self-selected usual-care comparator using data from the products platform. Among the three studies relying on user input data, one prospective study used self-selected limited app feature usage as a comparator, and two retrospective studies used either propensity score-matched usual care from registry data or propensity-adjusted comparisons across therapeutic modules. Detailed descriptions of study design, evidence levels, and comparator types are provided in Supplementary Table 3.

**Fig. 5:**
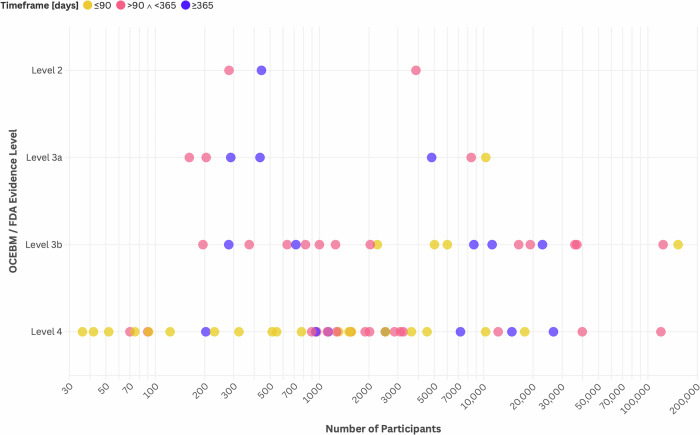
Distribution of evidence level and study size (number of participants) in identified studies (Bubble color represents the duration (in days) over which application usage was monitored).

Study sample sizes varied considerably (Fig. 5), with a median of 1529 participants (mean: 10,666). Retrospective studies generally had larger sample sizes (median: 2027; mean: 12,888) compared to prospective studies (median: 633; mean: 3999). Study durations ranged broadly from 14 days to 5 years, with a median duration of 180 days (mean: 300 days). Short-term studies ( ≤ 90 days) comprised approximately 33% (24/72), and studies of intermediate duration (6 months–1 year) accounted for 44% (32/72). Long-term studies exceeding 1 year were less common, representing 22% (16/72) of the total.

## Discussion

This scoping review examined how RWD generated during routine use of patient-facing mHealth applications is being used to support effectiveness evaluation in peer-reviewed studies. Using published research as a proxy for current evidence-generation practices, we mapped types of mHealth applications assessed with RWD, the data categories and types employed, and the range of the study designs and respective evidence levels used. We observed substantial variation in RWD characteristics and study designs, highlighting current patterns and gaps in the integration of RWD into effectiveness assessments across the field.

In 72 included studies, we identified 61 unique mHealth applications using naturally emerging RWD. These were unevenly distributed across medical specialties and application types. The observed concentration of applications in mental health (*n* = 19) and endocrine/metabolic disorders (*n* = 18) suggests that these domains may be more amenable to current RWD collection and evaluation practices. In mental health, 89% of applications (17/19) used data actively entered by the user, most commonly via in-app symptom surveys (e.g., PHQ-9, ISI), aligning with the clinical need to capture subjective patient perceptions such as mood, anxiety, or sleep quality^34^. In metabolic disorders, 50% (9/18) employed device-generated data, e.g., from connected sensors such as continuous glucose monitors and Bluetooth-enabled scales, supporting the collection of objective physiological data^30^. Interestingly, musculoskeletal and mental health apps rarely use wearable sensor data despite demonstrated potential to enhance objective outcome assessments^31^. Notably, no diagnostic application using RWD was identified, which may reflect broader challenges in generating the type of high-quality RWD, such as structured clinical observations or confirmatory testing, needed to evaluate diagnostic performance^23,35^.

The characteristics of RWD employed also reflect opportunities and constraints. User input data was the most common data category, used in 71% of studies (51/72), reflecting how easily such data can be embedded into app interfaces. When only considering studies using in-app surveys (35/72), we found that more than a third (37%, 13/35) relied on non-validated instruments, which may limit comparability across interventions and complicate interpretation across studies. While PROMs offer valuable insights into subjective health states, their evidentiary value depends heavily on standardization and methodological rigor. In contrast, device-generated data from connected devices appeared in only 26% of studies (19/72), despite their potential to provide objective, high-frequency, and passive monitoring. Although especially useful in conditions with clear physiological markers, such as glucose levels in diabetes, wearable and sensor data are currently less frequently represented in domains like mental health, where emerging evidence suggests they could serve as proxies for clinical symptoms such as depressive episodes^31^. Only three hybrid studies (3/72) combined multiple RWD categories. However, integrating data from various categories can enhance the comprehensiveness and robustness of evaluations^36,37^. For instance, combining subjective PROMs with continuous glucose data has been shown to improve the precision and timeliness of diabetes management^38^. The rare use of system-generated data (5/72) underscores the lack of integration between health apps and healthcare systems. At the same time, the current fragmentation of data use reinforces the broader need to tailor evidence strategies to the capabilities and constraints of specific mHealth application types, rather than relying on uniform evaluation models^39^.

The distribution of study designs illustrates the range of methodological approaches currently used in published RWD-based mHealth evaluations. Evidence levels in this review reflect study design characteristics and should not be interpreted as supporting effectiveness inference. In practice, most studies relied on pre-post single-group designs without comparators, which correspond to lower evidence levels within our classification framework adapted for the scope of this review from the OCEBM^29^ and FDA RWE framework^23^ (Supplementary Note 3). Only a minority employed quasi-experimental approaches or included real-world comparator groups, despite clear regulatory guidance encouraging the use of external or synthetic controls for RWE generation^40–42^. Notably, the few studies integrating clinical or claims data as comparators achieved higher levels of evidence. For example, one prospective cohort study evaluating an app designed to reduce emergency department admissions used routine EHR data to construct a concurrent usual-care comparator, raising its evidence level from 3b to 2a^39^. Importantly, among studies using system-generated health data, a small number employed randomized or pragmatic randomized designs, suggesting compatibility between controlled trial methodologies and real-world data. This underscores the value of linked, longitudinal data in improving study quality. Even among studies that collect substantial volumes of RWD, design features such as the absence of predefined outcomes, short study durations, or passive observational designs constrain the types of inferences these studies are able to support. While retrospective designs are cost-efficient and often feature large samples, they typically lack the planning necessary to control for bias. To advance evidentiary standards, future research should incorporate more rigorous comparator strategies, including pragmatic randomized controlled trials^43^ or advanced quasi-experimental methods like interrupted time series or difference-in-differences analyses^24^.

mHealth applications’ inherent capability to continuously collect RWD uniquely positions them for dynamic, ongoing effectiveness monitoring on-market, aligning well with evolving health technology assessment frameworks^24,44^. However, this potential is not yet widely reflected in current evaluation designs. Although 44% of studies (32/72) employed continuous or continual data collection, the majority (56%, 40/72) relied on intermittent, periodic, or one-off approaches and only 22% of studies (16/72) featured a long-term follow-up. This indicates that most evaluations still treat app data as static snapshots rather than sources of real-time, evolving insight, highlighting a broader gap in the sustained assessment of effectiveness over time.

A key strength of this review is its transparent approach to mapping how naturally emerging RWD is currently used to evaluate patient-facing mHealth applications. We applied predefined, literature-based frameworks to classify application types (NICE Digital Health Technology evidence standards^33^), RWD categories (adapted from Swift et al.^45^), and study design characteristics (based on the Oxford Centre for Evidence-Based Medicine^29^ and FDA RWE guidance^23^). This structured methodology, combined with a piloted and expert-informed data extraction process, enabled a detailed cross-sectional overview of current evaluative practices. The synthesis highlights methodological gaps and provides a foundation for aligning future evidence generation with evolving regulatory expectations. All extracted data is available in Supplementary Tables 2 and 3 to support transparency and reproducibility.

This review has several limitations. Only peer-reviewed, English-language publications were included, which may have introduced language bias and excluded gray literature. Despite iterative refinement of search strings, the absence of standardized terminology in digital health may have led to missed studies. Our focus on effectiveness outcomes reflects regulatory priorities but excludes other important aspects such as safety, cost-effectiveness, or user experience, which warrant further investigation. Although we searched three major medical and multidisciplinary databases, we did not include engineering-focused databases such as IEEE Xplore or the ACM Digital Library. As a result, some technically oriented studies without a primary clinical effectiveness focus may not have been captured. Study selection involved initial calibration by two reviewers on a random sample, followed by independent screening with regular consensus checks; although inter-rater reliability was not formally calculated, this consensus-based approach aligns with best practice in scoping reviews. Data extraction and study classification were conducted by a single reviewer. While this approach is acceptable for scoping reviews, it may increase the risk of misclassification, particularly given the multi-dimensional nature of our framework (mHealth application category, RWD source, study design, and evidence level). To mitigate this risk, data extraction was piloted by two reviewers with 10 studies, classification rules were predefined, and regular consensus discussions were held within the review team to resolve ambiguities. Nevertheless, some degree of subjective interpretation cannot be fully excluded. In addition, some included studies evaluated mHealth applications delivered under trial-like/highly structured care settings. Although these outcomes were generated via routine in-app functionality and classified as RWD in this review, these hybrid contexts are not fully naturalistic and may limit generalisability. Finally, because traditional frameworks like OCEBM do not fully capture how digital health tools generate and use RWD, we combined it with the FDA’s RWE framework. The combination of OCEBM and FDA RWE frameworks was necessary to address the unique features of digital health data, but further tailored evaluation standards are needed. Our evidence-level classification is applied at the level of the individual RWD-based effectiveness study and is design-centred it does not constitute a comprehensive reassessment of the overall clinical effectiveness or regulatory evidence base for the underlying technologies. The absence of widely accepted evaluation standards reinforces the need for baseline mapping of current practices, which this review provides.

Although support for RWE in policy is growing, its adoption in digital health research remains limited. While study volume has increased since 2021, the overall number of evaluations using RWD to generate evidence remains modest and highlights a gap between regulatory ambition and implementation. This reflects ongoing challenges in translating policy ambitions into research practice^46^. Most studies relied on limited data types, short follow-up periods, and non-comparator designs, with only a few integrating external data sources or using designs aligned with higher evidence levels. These patterns may reflect a combination of factors, including, but not limited to, the continued predominance of trial-centered evaluation practices in some regulatory contexts^47^ and the substantial heterogeneity of existing digital health evaluation frameworks^48^. To advance the field, adaptable but clear regulatory frameworks and improved access to clinical and claims data will be essential^49,50^. In this context, the forthcoming European Health Data Space may play a pivotal role by facilitating secure access to high-quality clinical data from routine care, thus enabling more rigorous and integrated real-world evidence generation^51^. Importantly, future mHealth evidence-generation strategies may benefit from stronger integration of comparator designs, continuous data capture, and multi-source RWD linkage. These steps will enhance the credibility and impact of mHealth applications in real-world care. To translate these system-level challenges into practical next steps for evidence generation, technology design, and policy, we summarize key implications in Box 1.

Box 1 Practical implications for advancing RWE-based evaluation of mHealth **Evidence generation**

- Future studies could increasingly move from predominantly single-group pre–post designs toward comparator-based and quasi-experimental approaches, including pragmatic randomized designs where feasible.
- Greater use of validated PROMs may help with subjective outcomes to support reliability and comparability across studies.
- The longitudinal nature of mHealth data offers opportunities for sustained effectiveness monitoring rather than short-term snapshot evaluations.

**Technology design and data infrastructure**

- Expand the use of device-generated and passive sensor data, particularly beyond metabolic indications and into domains such as mental health.
- Design applications with interoperability and future linkage to clinical and administrative data as a core requirement.

**Health system and regulatory context**

- Strengthen technical and governance frameworks for secure app–EHR/claims data linkage to enable higher-quality real-world evidence generation.
- Support fit-for-purpose RWE standards that reflect the iterative and data-rich nature of mHealth and incentivize longitudinal, comparator-based post-market evaluation.

## Methods

We conducted a scoping review following the framework by Tricco et al^52^. to map how RWD are used in peer-reviewed studies evaluating the effectiveness of mHealth applications and to identify key themes and research gaps. This format was chosen to characterise current evaluation practices focusing on RWD characteristics, study designs and applied evidence frameworks, rather than to estimate the intervention effectiveness or establish causal effects. The PRISMA-ScR checklist^53^ guided reporting (Supplementary Table 1), and the protocol was registered prospectively on the Open Science Framework (https://osf.io/3f5ur/).

### Search strategy

A three-step search strategy, based on Joanna Briggs Institute (JBI) guidelines^54–56^, was implemented. A preliminary search in PubMed identified key articles related to mHealth, RWD use, and effectiveness assessment. Using 11 key studies as a starting point, a search strategy was developed, refined with an expert librarian, and adapted for PubMed, Scopus, and Web of Science. These databases were selected to provide comprehensive coverage of biomedical, clinical, and applied digital health research. The search strings combined three core concept blocks: (1) mHealth and digital health applications (e.g., “mHealth app”, “digital health application”), (2) effectiveness and performance outcomes (e.g., “effectiveness”, “clinical outcome”, MeSH terms for outcome assessment), and (3) real-world data concepts (e.g., “real-world data”, “real-world evidence”, “electronic health records”, “claims data”, “registry”). The final searches for all databases were executed on November 29, 2024. Backward and forward citation tracking supplemented database searches. No direct contact with study authors was undertaken to identify additional sources. The complete electronic search strings with all Boolean operators and database-specific search string adaptations are provided in Supplementary Note 1.

### Eligibility criteria

We defined eligibility criteria using the PCC (Population, Concept, Context)^54^ framework. The population included studies evaluating mHealth applications designed for independent use by patients to improve health or clinical outcomes. Applications intended for use primarily by healthcare professionals or caregivers were excluded.

The concept focused on the use of naturally emerging RWD to support the effectiveness-related evaluation of these applications. Naturally emerging RWD were defined as data produced during routine app use through standard in-app functionality, whether actively entered by users, passively captured by sensors or integrated from health-system sources, without study-specific instruments.

The context was real-world care settings, for example, home or routine clinical practice. Study-specific recruitment and participant onboarding were permitted. Inclusion was based on outcome data captured through standard app functions during routine use, as well as routinely collected healthcare system data generated in the context of real-world application use, and excluded outcome data collected via external, study-specific research instruments. We focused on patient-facing mHealth applications that support health improvement or clinical outcomes, specifically those used independently by adults for self-management and health information tracking. To maintain this focus, we excluded apps developed primarily for productivity or efficiency enhancement, as well as those intended solely for educational purposes or for patient-provider communication.

The publication timeframe (January 2007 to November 2024) was selected to align with the introduction of modern touchscreen smartphones and the resulting emergence of mHealth applications as a distinct category of digital health technologies. Restriction to peer-reviewed literature was applied to ensure a minimum level of methodological reporting quality and scientific scrutiny. Studies published in English were included due to resource constraints for high-quality translation. A minimum sample size threshold of 30 participants was applied to exclude pilot-level feasibility studies and to ensure inclusion of studies with a basic level of empirical stability for mapping evaluation practices.

Additional application characteristics were specified to guide consistent inclusion and exclusion decisions, particularly regarding intended use and functionality. Full criteria are detailed in Table 4.

**Table 4:** Eligibility criteria based on the PCC framework (Population, Concept, Context)

Criteria	Inclusion	Exclusion
Population	Studies with a focus on mHealth applications designed for independent use by patients in real-world settings Study participants 18 years or older	Studies focusing on mHealth applications intended to be used by HCPs or caregivers Studies focusing on mHealth applications designed to collect data for HCPs without an interface for patients or without an active role for the patient in data collection and usage Study participants under 18 years
Concept	Studies evaluating the effectiveness of mHealth applications in achieving their intended health outcomes using RWD Preliminary efficacy studies were included, provided they were conducted with diagnosed patients	Studies evaluating user experience, usability, or acceptance of mHealth applications Studies on economic value/cost savings Preliminary efficacy, validation or feasibility studies with fewer than 30 participants
Context	Studies conducted in real-world care settings (e.g., at home, in the community or clinical practice) Studies acquiring data within routine use of the application using standard in-app functionalities (e.g., sensor tracking, app usage logs, in-app symptom tracking, in-app surveys), without modification of the core application functionality for research purposes. Study-specific recruitment was permitted Studies using routinely collected healthcare system data generated in the context of real-world application use (e.g., claims data, electronic health records, pharmacy dispensing data, or hospital utilization)	Study settings not reflecting real-world use of the device (e.g., simulated or laboratory-based settings) Studies using applications modified specifically for research Studies in which outcome data were collected primarily through external, study-specific instruments or platforms explicitly described as separate from the routine app environment, including separate survey tools, email questionnaires, or non–app-based assessments. Studies where data is generated primarily through professional-grade equipment in structured clinical environments (e.g., hospital at home), even if supplemented by consumer wearables
Application Characteristics/Scope	mHealth applications, defined as smartphone or web-based health applications on mobile devices (e.g., smartphones and tablets) mHealth applications may operate standalone or in combination with (external) sensors mHealth applications must be intended to improve health outcomes, support behavior change, or assist with chronic disease self-management	mHealth applications designed solely for passive information delivery (e.g., educational apps without tracking, personalization, or intervention components) mHealth applications limited to patient–provider communication (e.g., secure messaging platforms, SMS-based triage, or remote consultation tools) that do not generate patient-entered or device-captured data during routine use and do not provide a patient-facing data interface
Other Criteria	Peer-reviewed empirical studies using quantitative, qualitative, or mixed methods Studies published between January 2007 and November 2024 Language of publication: English	Reviews, books or non-empirical research (e.g., systematic reviews, letters, opinion pieces, theoretical papers) Study protocols and non-peer-reviewed articles (e.g., preprints, unpublished trial data, conference proceedings) Studies published before 2007 Studies published in languages other than English

### Study selection

Search results were imported into Covidence (Veritas Health Innovation) for automated duplication removal, title, abstract, and full-text screening. Two reviewers (S.B. and S.Ge.) independently screened a random sample of 20 studies to calibrate inclusion decisions. Following calibration, both reviewers independently screened all titles, abstracts, and full texts at each stage against the predefined eligibility criteria. Disagreements were resolved through consensus meetings. If consensus could not be reached, a third author was consulted to make a final determination. The study selection process is summarized in the PRISMA flow diagram (Fig. 1).

### Data extraction and analysis

Two reviewers (S.B. and S.Ge.) developed a standardized data extraction form (Google Sheets), piloted it with 10 studies, and iteratively refined it based on included studies and an expert interview with an mHealth scientific director. In line with JBI guidelines^54^, final data extraction was conducted by one reviewer (S.Ge.), due to resource constraints. To ensure consistency and accuracy, regular discussions were held with the review team to resolve uncertainties and verify data interpretation. Data synthesis was performed in Microsoft Excel (Microsoft Office, 2019).

The following variables were extracted:

- Study details, including study title, authorship, DOI, year of publication, journal, country of participants, recruitment context (routine users vs. research-recruited), number of participants, and timeframe of data analysis (in days).
- Application details, including application name, medical specialty, primary intended app purpose, mHealth category, and potential medical device status, including whether formal regulatory information was publicly available.
- Data Details, including measured health outcome (study endpoint), mode of data collection (active vs. passive), RWD category (user input, device-generated, system-generated), data type (e.g. in-app surveys, connected devices, app engagement, EHR/claims data), use of validated in-app instruments, data collection frequency and frequency specifications, as well as up to two primary parameters and their associated measurement tools.
- Study Design Details, including study design classification based on the adapted OCEBM/FDA RWE framework, interventional versus observational intent, presence of randomization, temporal orientation (prospective vs. retrospective), mechanisms of outcome comparison (e.g., intra-individual, intergroup), type and nature of comparator or control condition, and assigned evidence level.
- Interpretive Study Design Summary, consisting of a structured free-text description of how real-world data were used in each study to evaluate the intended application purpose.

**Fig. 6:**
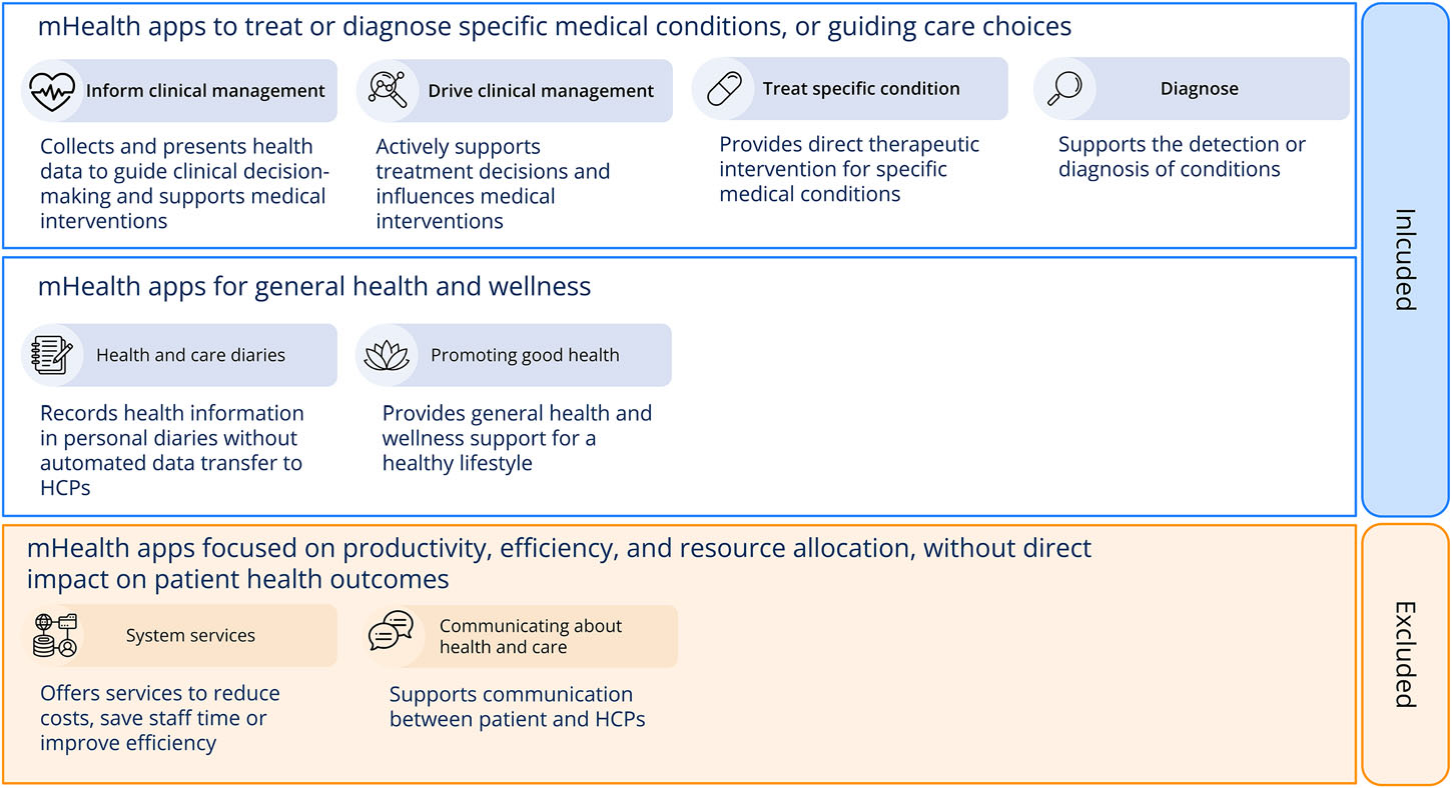
Classification of patient-used mHealth applications to improve health outcomes based on the mHealth taxonomy by Olla & Shimskey, as well as the NICE evidence standard framework. MHealth applications included in the review are marked in blue. *System services* and *communicating about health and care* marked in orange are outside the scope of this review. HCPs = Healthcare professionals.

To classify the mHealth applications in categories, we used a combined framework based on the mHealth taxonomy by Olla & Shimskey^32^ and the NICE evidence standards framework^33^ (see Fig. 6), which stratifies tools based on their intended medical function. Applications were categorized according to their primary intended function, even when they featured multiple functionalities.

RWD were categorized using a refined version of the framework proposed by Swift et al.^45^, tailored to mobile app–based interventions. The final taxonomy comprises three categories: User Input Data, Device-Generated Data, and System-Generated Data. User input data included in-app surveys capturing patient-reported outcomes, manually entered vital stats and event tags; device-generated data included connected devices, app engagement, task performance, and mobile device data like activity metrics; and system-generated data included admission/discharge reports, procedures, vital stats or medication variables derived from electronic health records and claims data. Details on the taxonomy adaptation process and category definitions are provided in Supplementary Note 2.

To assess study design, we applied the Oxford Centre for Evidence-Based Medicine (OCEBM) 2011 framework^29^ in combination with the FDA Real-World Evidence framework^23^, which together informed our classification of levels of evidence for the specific scope of this review. The combined evidence grading approach is described in Supplementary Note 3. Although mHealth applications constitute the unit of analysis in this review, the evidence grading is applied at the level of the individual effectiveness study and is specific to our research scope. Classification was based on structural study design characteristics, including interventional versus observational intent, presence or absence of randomization, temporal orientation (prospective versus retrospective), mechanisms of outcome comparison (intra-individual versus intergroup), and the presence and type of external comparator. No formal risk-of-bias appraisal was conducted, and the assigned evidence levels do not represent a comprehensive appraisal of the overall clinical effectiveness or regulatory evidence base of the respective technologies.

All analytical categories were operationalized during piloting of the data extraction form and assigned directly at the point of data extraction. When methodological reporting was ambiguous (e.g., whether an in-app survey was pre-existing or study-specific), conservative coding decisions were applied strictly based on explicit descriptions in the original publications. For in-app survey instruments, validation status was classified as validated when the study explicitly reported use of an established, previously validated PROM. Instruments developed specifically for the application or study without reference to prior validation were classified as not validated. No independent reassessment of psychometric properties or digital-format validation was performed. No imputation of missing variables was performed. The extracted study-level variables were then used as grouping variables for quantitative aggregation in Tables 1–3 and Figs. 2–5, and the narrative synthesis was structured iteratively around the predefined research questions and the resulting distributions.

Descriptive statistics and narrative synthesis identified key patterns and trends in the extracted data. Data are presented in tables and graphical formats where appropriate. Percentages are rounded to one decimal place where applicable, and continuous variables are summarized using medians (with means reported for descriptive reference). Unless otherwise stated, all proportions reported in the Results refer to the total number of studies included (*N* = 72). Studies using hybrid data sources (e.g., user input combined with device-generated data) are counted in all applicable RWD categories; therefore, percentages across categories may exceed 100%.

This structured approach enabled comparability across studies. The full list of included studies and extracted data is provided in Supplementary Tables 2 and 3. No additional data were sought from the original study authors.

### Critical appraisal of included sources

Consistent with the objectives of this scoping review and with PRISMA-ScR and JBI guidance, no formal critical appraisal or risk-of-bias assessment of individual studies was conducted. The aim of this review was to map the extent, nature, and methodological characteristics of published evaluations using real-world data in mHealth, rather than to assess intervention effects or to compare the internal validity of individual studies. Moreover, the included evidence base comprised highly heterogeneous study designs (ranging from single-group pre–post studies to pragmatic randomized trials), app types, and data sources, which would have required multiple, design-specific appraisal tools and would not have supported a unified quality scoring approach. Accordingly, critical appraisal results were not used to inform study inclusion or data synthesis. Instead, methodological heterogeneity was addressed descriptively through explicit classification of study design features and evidence levels using the adapted OCEBM–FDA real-world evidence framework.

### Data synthesis and summarization

Charted data were synthesized using descriptive quantitative methods and structured narrative synthesis. Study-level variables were aggregated using absolute counts and proportions to summarize distributions across application types, real-world data categories, and study design features. No statistical pooling or meta-analysis was undertaken, as the objectives of the review were descriptive and the included studies were highly heterogeneous with respect to interventions, outcomes, and data generation modalities. Findings were summarized across predefined analytical groupings aligned with the review questions and are presented in tabular and graphical form. The narrative synthesis was developed iteratively from the quantitative distributions to contextualize observed patterns, gaps, and methodological tendencies in the use of real-world data for mHealth evaluation.

## Supplementary information

Supplementary Information

## Data Availability

All data generated and analyzed during this study are included in the article and its Supplementary Information.
