# Supplementary material for: A scoping review on using real-world data to evaluate the effectiveness of mHealth applications
Source: NPJ Digit Med. 2026 Apr 8;9:309. doi: 10.1038/s41746-026-02562-0 (PMC13068921; doi:10.1038/s41746-026-02562-0)
Supplement: Supplementary file 1 — Supplementary Information [file 41746_2026_2562_MOESM1_ESM.pdf]

A Scoping Review on Using Real-World Data to Evaluate the Effectiveness of mHealth Applications

Supplementary Information

This appendix formed part of the original submission and has been peer reviewed.

Table of contents

Table of contents 1

Supplementary Information Note 1: Complete Search Strings 2

Supplementary Information Note 2: Taxonomy for Classification of Real-World Data 7

Supplementary Information Note 3: Framework for Assessment of Evidence Level 9

Supplementary Information Table 1: PRISMA-ScR Checklist 12

Supplementary Information Table 2: Overview of full Study Details, Application Details and Data Details for all included studies 15

Supplementary Information Table 3: Overview of full Study Design Details including Evidence Levels (adapted OCEBM/FDA RWE) and Key Sample Descriptors for all included studies 22

OSF-Protocol Deviations 36

References 37

Supplementary Information Note 1: Complete Search Strings

Search date: 29.11.2024

PubMed - hits: 4,611

(("app"[tiab] OR "mHealth app"[tiab] OR "mobile health app"[tiab] OR "health app"[tiab] OR "digital health app"[tiab] OR "Digital Health Applications"[tiab] OR "digital health applications"[tiab] OR "smartphone app"[tiab] OR "smartphone-based"[tiab] OR "smartphone based"[tiab] OR "web-based app"[tiab] OR "smartphone-based home exercise program"[tiab] OR "mobile phone app"[tiab] OR "behavior change"[tiab] OR "behaviour change"[tiab] OR "behavior change"[tiab] OR "digital behavior change"[tiab] OR "digital intervention"[tiab] OR "digital health intervention"[tiab] OR "digital cognitive-behavioral therapy"[tiab] OR "digital cognitive-behavioural therapy"[tiab] OR "cognitive-behavioral therapy"[tiab] OR "cognitive-behavioural therapy"[tiab] OR "dCBT"[tiab] OR "CBT"[tiab] OR "digital home exercise"[tiab] OR "digital therapy"[tiab] OR "blended care intervention"[tiab] OR "blended-care intervention"[tiab] OR "blended-care treatment"[tiab] OR "remote smartphone monitoring"[tiab] OR "smartphone monitoring"[tiab] OR "digital monitoring"[tiab] OR "remote monitoring"[tiab] OR "patient monitoring"[tiab] OR "remote health assessment*"[tiab] OR "point-of-care diagnostic"[tiab] OR "point-of-care diagnostic app*"[tiab] OR "point of care app*"[tiab] OR "point-of-care device"[tiab] OR "diagnostic app*"[tiab] OR "diagnostic application"[tiab] OR "AI diagnostic tool"[tiab] OR "self-triage"[tiab] OR "DiGA"[tiab] OR "Digital Home Exercise Program"[tiab] OR "smartphone* AND wearable*"[tiab] OR "Digital health Treatment program"[tiab] OR "digital health solution*"[tiab] OR "Artificial Intelligence App*"[tiab] OR "personal digital device"[tiab] OR "portable device"[tiab] OR "point-of-care settings"[tiab] OR "smartphone-based home exercise program"[tiab]))

AND (("effectiveness"[tiab] OR "efficacy"[tiab] OR "effect"[tiab] OR "outcome*"[tiab] OR "health outcome*"[tiab] OR "patient outcome*"[tiab] OR "therapy outcome*"[tiab] OR "treatment outcome*"[tiab] OR "intervention outcome*"[tiab] OR "care outcome*"[tiab] OR "therapeutic outcome*"[tiab] OR "clinical outcome*"[tiab] OR "impact"[tiab] OR "intervention impact"[tiab] OR "app engagement"[tiab] OR "app usage"[tiab] OR "diagnostic performance"[tiab] OR "diagnostic accuracy"[tiab] OR "accurac*"[tiab] OR "performance"[tiab] OR "logging-activity"[tiab] OR "predictor for treatment outcomes"[tiab] OR "pain intensity"[tiab] OR "reliability"[tiab] OR "symptom improvement"[tiab] OR "therapy adherence"[tiab] OR "disease severity"[tiab] OR "patient engagement"[tiab] OR "speed tapping"[tiab] OR "phonation"[tiab] OR "app utilization"[tiab] OR "duration of use"[tiab] OR "topics assessed"[tiab] OR "step count measurement"[tiab] OR "treatment outcome"[MeSH Terms] OR "Outcome Assessment, Health Care"[MeSH] OR "Clinical Effectiveness"[MeSH] OR "Comparative Effectiveness Research"[MeSH] OR "Evidence-Based Medicine"[MeSH]))

AND (("real-world data"[tiab] OR "real world data"[tiab] OR "RWD"[tiab] OR "real-world evidence"[tiab] OR "real-world evidence study"[tiab] OR "real world evidence"[tiab] OR "real-world evidence study"[tiab] OR "real world evidence study"[tiab] OR "RWE"[tiab] OR "registry"[tiab] OR "registry data"[tiab] OR "claims"[tiab] OR "claims data"[tiab] OR "EHR"[tiab] OR "electronic health record*"[tiab] OR "pragmatic randomized controlled trial"[tiab] OR "pragmatic randomised controlled trial"[tiab] OR "pRCT"[tiab] OR "observational study"[tiab] OR "observational cross-sectional study"[tiab] OR "observational data"[tiab] OR "real-world prescription data"[tiab] OR "real-world users"[tiab] OR "retrospective cohort study"[tiab] OR "retrospective observational study"[tiab] OR "validation study"[tiab] OR "Real World NHS Evaluation"[tiab] OR "surrogate marker"[tiab] OR "surrogate parameter"[tiab] OR "biomarker"[tiab] OR "sensor data"[tiab] OR "wearable sensor"[tiab] OR "wearable device"[tiab] OR "biosensor"[tiab] OR "digital sensor"[tiab] OR "Observational Study"[MeSH] OR "Pragmatic Clinical Trials as Topic"[MeSH] OR "Surrogate Endpoints"[MeSH]))

AND hasabstract

NOT (Review[pt] OR "review"[tiab] OR "editorial"[pt] OR "opinion article"[tiab] OR "critical view"[tiab] OR "opinion paper"[tiab] OR "perspective article"[tiab] OR "perspective paper"[tiab])

Scopus - hits 2.904

(TITLE-ABS-KEY ((("app" OR "mHealth app" OR "mobile health app" OR "health app" OR "digital health app" OR "Digital Health Applications" OR "digital health applications" OR "smartphone app" OR "smartphone-based" OR "smartphone based" OR "web-based app" OR "smartphone-based home exercise program" OR "mobile phone app" OR "behavior change" OR "behaviour change" OR "bevahior change" OR "digital behavior change" OR "digital intervention" OR "digital health intervention" OR "digital cognitive-behavioral therapy" OR "digital cognitive-behavioural therapy" OR "cognitive-behavioral therapy" OR "cognitive-behavioural therapy" OR "dCBT" OR "CBT" OR "digital home exercise" OR "digital therapy" OR "blended care intervention" OR "blended-care intervention" OR "blended-care treatment" OR "remote smartphone monitoring" OR "smartphone monitoring" OR "digital monitoring" OR "remote monitoring" OR "patient monitoring" OR "remote health assessment*" OR "point-of-care diagnostic" OR "point-of-care diagnostic app*" OR "point of care app*" OR "point-of-care device" OR "diagnostic app*" OR "diagnostic application" OR "AI diagnostic tool" OR "self-triage" OR "DiGA" OR "Digital Home Exercise Program" OR "smartphone* OR wearable*" OR "Digital health Treatment program" OR "digital health solution*" OR "Artificial Intelligence App*" OR "personal digital device" OR "portable device" OR "point-of-care settings" OR "smartphone-based home exercise program")))) AND (TITLE-ABS-KEY ((("effectiveness" OR "efficacy" OR "effect" OR "outcome*" OR "health outcome*" OR "patient outcome*" OR

"therapy outcome*" OR "treatment outcome*" OR "intervention outcome*" OR "care outcome*" OR "therapeutic outcome*" OR "clinical outcome*" OR "impact" OR "intervention impact" OR "app engagement" OR "app usage" OR "diagnostic performance" OR "diagnostic accuracy" OR "accurac*" OR "performance" OR "logging-activity" OR "predictor for treatment outcomes" OR "pain intensity" OR "reliability" OR "symptom improvement" OR "therapy adherence" OR "disease severity" OR "patient engagement" OR "speed tapping" OR "phonation" OR "app utilization" OR "duration of use" OR "topics assessed" OR "step count measurement" OR "treatment outcome" OR "Outcome Assessment, Health Care" OR "Treatment Outcome" OR "Clinical Effectiveness" OR "Comparative Effectiveness Research" OR "Evidence-Based Medicine")))) AND (TITLE-ABS-KEY ((("real-world data" OR "real world data" OR "RWD" OR "real-world evidence" OR "real-world evidence study" OR "real world evidence" OR "real-world evidence study" OR "real world evidence study" OR "RWE" OR "registry" OR "registry data" OR "claims" OR "claims data" OR "EHR" OR "electronic health record*" OR "pragmatic randomized controlled trial" OR "pragmatic randomised controlled trial" OR "pRCT" OR "observational study" OR "observational cross-sectional study" OR "observational data" OR "real-world prescription data" OR "real-world users" OR "retrospective cohort study" OR "retrospective observational study" OR "validation study" OR "Real World NHS Evaluation" OR "surrogate marker" OR "surrogate parameter" OR "biomarker" OR "sensor data" OR "wearable sensor" OR "wearable device" OR "biosensor" OR "digital sensor" OR "Observational Study" OR "Pragmatic Clinical Trials as Topic" OR "Surrogate Endpoints")))) AND NOT (review OR "review" OR "editorial" OR "opinion article" OR "critical view" OR "opinion paper" OR "perspective article" OR "perspective paper")

Additional Filters
“Subject area”: medicine, health professions, nursing, psychology

Web of Science - hits 3,307

TS=("app" OR "mHealth app" OR "mobile health app" OR "health app" OR "digital health app" OR "digital health application*" OR "Digital Health Applications" OR "smartphone app" OR "smartphone-based" OR "smartphone based" OR "web-based app" OR "smartphone-based home exercise program" OR "mobile phone app" OR "behavior change" OR "behaviour change" OR "bevahior change" OR "digital behavior change" OR "digital intervention" OR "digital health intervention" OR "digital cognitive-behavioral therapy" OR "digital cognitive-behavioural therapy" OR "cognitive-behavioral therapy" OR "cognitive-behavioural therapy" OR "dCBT" OR "CBT" OR "digital home exercise" OR "digital therapy" OR "blended care intervention" OR "blended-care intervention" OR "blended-care treatment" OR "remote smartphone

monitoring" OR "smartphone monitoring" OR "digital monitoring" OR "remote monitoring" OR "patient monitoring" OR "remote health assessment*" OR "point-of-care diagnostic" OR "point-of-care diagnostic app*" OR "point of care app*" OR "point-of-care device" OR "diagnostic app*" OR "diagnostic application" OR "AI diagnostic tool" OR "self-triage" OR "DiGA" OR "Digital Home Exercise Program" OR "smartphone* AND wearable*" OR "Digital health Treatment program" OR "digital health solution*" OR "Artificial Intelligence App*" OR "personal digital device" OR "portable device" OR "point-of-care settings" OR "smartphone-based home exercise program")
AND
TS=("effectiveness" OR "efficacy" OR "effect" OR "outcome*" OR "health outcome*" OR "patient outcome*" OR "therapy outcome*" OR "treatment outcome*" OR "intervention outcome*" OR "care outcome*" OR "therapeutic outcome*" OR "clinical outcome*" OR "impact" OR "intervention impact" OR "app engagement" OR "app usage" OR "diagnostic performance" OR "diagnostic accuracy" OR "accurac*" OR "performance" OR "logging-activity" OR "predictor for treatment outcomes" OR "pain intensity" OR "reliability" OR "symptom improvement" OR "therapy adherence" OR "disease severity" OR "patient engagement" OR "speed tapping" OR "phonation" OR "app utilization" OR "duration of use" OR "topics assessed" OR "step count measurement")
AND
TS=("real-world data" OR "real world data" OR "RWD" OR "real-world evidence" OR "real-world evidence study" OR "real world evidence" OR "real-world evidence study" OR "real world evidence study" OR "RWE" OR "registry" OR "registry data" OR "claims" OR "claims data" OR "EHR" OR "electronic health record*" OR "pragmatic randomized controlled trial" OR "pragmatic randomised controlled trial" OR "pRCT" OR "observational study" OR "observational cross-sectional study" OR "observational data" OR "real-world prescription data" OR "real-world users" OR "retrospective cohort study" OR "retrospective observational study" OR "validation study" OR "Real World NHS Evaluation" OR "surrogate marker" OR "surrogate parameter" OR "biomarker" OR "sensor data" OR "wearable sensor" OR "wearable device" OR "biosensor" OR "digital sensor")
AND PY=(2008-2024)
NOT
TS=("review" OR "editorial" OR "opinion article" OR "critical view" OR "opinion paper" OR "perspective article" OR "perspective paper")

Filter:
“Refine by research area”- selected categories: Health Care Scientists Services, Medical Informatics, General Internal Medicine, Psychiatry, Neuroscience Neurology, Psychology, Research Experimental Medicine, Biophysics, Oncology, Pharmacology Pharmacy, Surgery,

Rehabilitation, Endocrinology Metabolism, Nursing, Orthopedics, Nutrition Dietetics, Obstetrics Gynecology, Immunology, Geriatrics Gerontology, Respiratory System, Hematology, Infectious Diseases, Rheumatology, Sport Sciences, Radiology Nuclear Medicine Medical Imaging, Emergency Medicine, Substance Abuse, Gastroenterology Hepatology, Ophthalmology, Anesthesiology, Transplantation, Urology Nephrology, Physiology, Genetics Heredity, Medical Laboratory Technology, Integrative Complementary Medicine, Behavioural Science, Dermatology, Pathology, Allergy, Dentistry Oral Surgery Medicine

Supplementary Information Note 2: Taxonomy for Classification of Real-World Data

To classify real-world data (RWD) in our review, we began with the taxonomy by Swift et al.[1], selecting four categories most relevant to patient-facing mHealth applications: "Patient Reported," "Mobile Health," "Claims," and "Clinical."

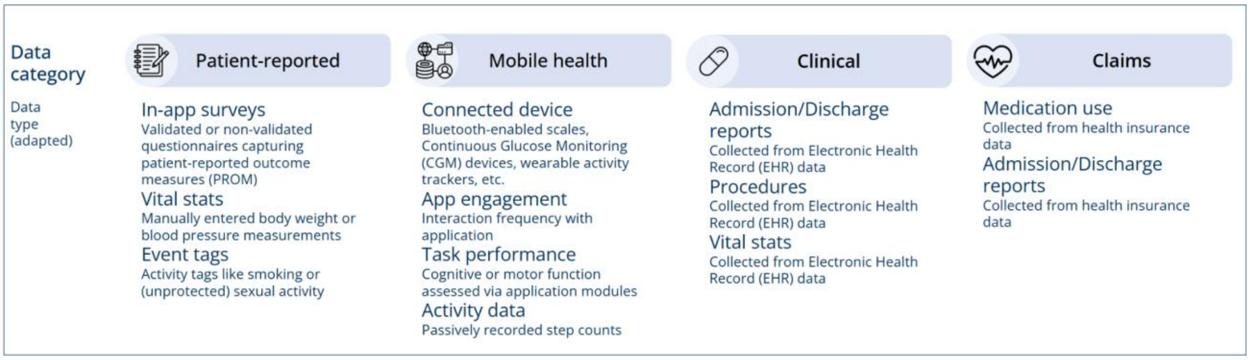

Figure 1 Initial taxonomy based on selected categories from Swift et al.[1], with expanded data types adapted for use in mHealth evaluation.

During piloting, we found that distinctions in data provenance, such as user-entered versus device-recorded data, were not clearly captured by the original framework. To address this, we reorganized our classification using two key dimensions in alignment with the FDA's Real-World Evidence (RWE) framework[2]: mode of data generation (active vs. passive) and point of origin (user, device, or external system).

The final taxonomy comprises three mutually exclusive categories:

- User Input Data (data actively entered by users via the app, e.g., symptom diaries),
- Device-Generated Data (data passively collected by sensors or mobile devices connected to the application, e.g., step counts), and
- System-Generated Data (data created by healthcare systems or professionals, e.g., EHRs, claims).

This approach accommodates overlapping data concepts by classifying them based on source and capture method, so the same metric (e.g., blood pressure) can be distinguished according to whether it is self-reported, sensor-derived, or extracted from records.

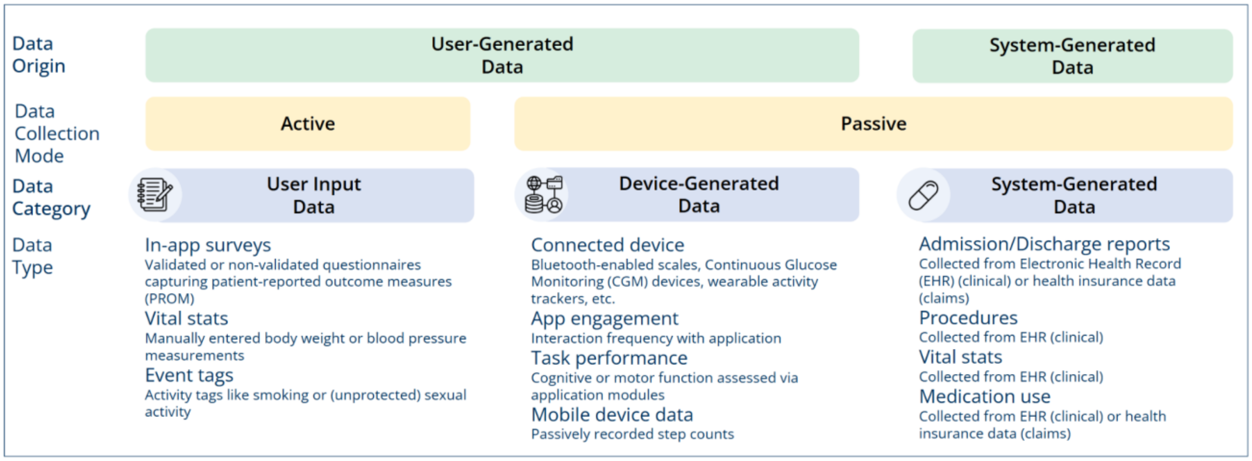

Figure 2 Taxonomy of Data Categories and Data Types used in this review

Figures 1 and 2 illustrate the shift from the original categories to this revised structure using the same data types. The final taxonomy supports consistent coding, analytical clarity, and synthesis across diverse study designs. While it draws on established frameworks and was empirically refined, it is designed as a practical tool for this review, not a universal classification system.

Supplementary Information Note 3: Framework for Assessment of Evidence Level

To evaluate the methodological rigour of included studies, we applied an adapted evidence framework combining the Oxford Centre for Evidence-Based Medicine (OCEBM) 2011 Levels of Evidence[3] with the FDA's framework for real-world evidence (RWE) evaluation[2]. This hybrid approach was selected to reflect the evolution of evidence generation in digital health, particularly the increasing relevance of real-world data (RWD) and quasi-experimental methods not fully captured by the traditional OCEBM hierarchy.

While the OCEBM framework remains foundational in evidence-based medicine, it predates the widespread use of digital and real-world methodologies. The FDA RWE guidance, in contrast, provides a contemporary view on the credibility of non-randomised studies for regulatory decision-making. By integrating both frameworks, we aimed to classify study designs in a way that better captures the heterogeneity and real-world relevance of digital health research.

Studies were categorised into Levels 2 to 4, with Levels 1 (systematic reviews) and 5 (expert opinion) excluded due to the focus on primary, empirical RWD studies. Figure 3 summarises the adapted classification and key design characteristics. Studies were grouped based on their design's capacity to support causal inference, with bias profiles assessed according to known methodological risks[4].

Evidence Level	Study Design Category	Study Design Description
1	Systematic Reviews & Meta-Analyses	Systematic reviews/meta-analyses of randomized controlled trials (RCTs) or high-quality cohort studies
2	Randomized Controlled Trials	Prospective randomized controlled trials (RCTs), including pragmatic clinical trials (PCTs)
	Prospective Cohort Studies	Prospective cohort studies with concurrent (same-time) controls (non-randomized)
3a	Quasi-Experimental RWE Designs	Interrupted time series (ITS), Difference-in-Differences (DiD), Regression Discontinuity (RDD), and studies with historical or retrospectively matched control
3b	Retrospective Cohort Studies	Retrospective observational cohort studies without control (Intergroup comparison)
4	Pre-Post Single-Arm Studies	Single-cohort, observational studies without a control group (pre-post comparison)
5	Mechanism-Based/ Expert Opinions	Mechanism-based reasoning, narrative reviews, editorials, expert opinions

Figure 3 Classification of Study Designs and Evidence Levels used in this review based on OCEBM Levels of Evidence and the Framework for FDA`s Real-World Evidence Program. Level 1 and 5 are outside the scope of this framework (marked orange).

We structured the classification to reflect how evidence quality improves incrementally through the addition of key design elements:

At Level 4, single-arm pre-post studies measure outcomes before and after an intervention in the same cohort without a comparator. While common in digital health implementation studies, they are vulnerable to maturation, history, and regression to the mean biases, limiting their causal interpretability[5]. The absence of a comparator group precludes control for external factors and interindividual variation.

Level 3b includes retrospective cohort studies with an intergroup comparison. These introduce a comparison group but lack prospective design and often use non-concurrent or poorly matched controls, increasing risk of selection, confounding, and information bias[6]. The design does not inherently account for temporal effects or unmeasured confounders.

Level 3a captures quasi-experimental designs - including interrupted time series (ITS), difference-in-differences (DiD), regression discontinuity (RDD), and retrospective matched-control studies. These designs offer structured attempts to emulate randomisation and improve causal inference using statistical control for time and confounding. However, they remain susceptible to residual selection bias and cannot fully eliminate unmeasured confounding[7].

Level 2 includes prospective cohort studies and (pragmatic) randomised controlled trials (RCTs). Prospective cohort studies with concurrent non-randomised comparators improve on lower-level designs by mitigating timing-related and recall biases but still face risks of confounding and attrition. RCTs provide the highest degree of internal validity through the minimisation of both measured and unmeasured confounders and serve as the reference standard in clinical research[8].

This tiered classification reflects not only the number and strength of design features but also their ability to address specific sources of bias. In doing so, it enables structured comparison across diverse real-world mHealth evaluations.

Supplementary Information Table 1: PRISMA-ScR Checklist

Preferred Reporting Items for Systematic reviews and Meta-Analyses extension for Scoping Reviews[9]

SECTION	ITEM	PRISMA-ScR CHECKLIST ITEM[9]	REPORTED ON PAGE #
**TITLE**			
Title	1	Identify the report as a scoping review.	1
**ABSTRACT**			
Structured summary	2	Provide a structured summary that includes (as applicable): background, objectives, eligibility criteria, sources of evidence, charting methods, results, and conclusions that relate to the review questions and objectives.	1
**INTRODUCTION**			
Rationale	3	Describe the rationale for the review in the context of what is already known. Explain why the review questions/objectives lend themselves to a scoping review approach.	2-3
Objectives	4	Provide an explicit statement of the questions and objectives being addressed with reference to their key elements (e.g., population or participants, concepts, and context) or other relevant key elements used to conceptualize the review questions and/or objectives.	3-4
**METHODS**			
Protocol and registration	5	Indicate whether a review protocol exists; state if and where it can be accessed (e.g., a Web address); and if available, provide registration information, including the registration number.	13
Eligibility criteria	6	Specify characteristics of the sources of evidence used as eligibility criteria (e.g., years considered, language, and publication status), and provide a rationale.	Table 5
Information sources*	7	Describe all information sources in the search (e.g., databases with dates of coverage and contact with authors to identify additional sources), as well as the date the most recent search was executed.	13
Search	8	Present the full electronic search strategy for at least 1 database, including any limits used, such that it could be repeated.	Supplementary Information – Note 1
Selection of sources of evidence†	9	State the process for selecting sources of evidence (i.e., screening and eligibility) included in the scoping review.	15, Table 5

Data charting process‡	10	Describe the methods of charting data from the included sources of evidence (e.g., calibrated forms or forms that have been tested by the team before their use, and whether data charting was done independently or in duplicate) and any processes for obtaining and confirming data from investigators.	16
Data items	11	List and define all variables for which data were sought and any assumptions and simplifications made.	Supplementary Information – Table 2, Table 4
Critical appraisal of individual sources of evidence§	12	If done, provide a rationale for conducting a critical appraisal of included sources of evidence; describe the methods used and how this information was used in any data synthesis (if appropriate).	Supplementary Information – Note 3
Synthesis of results	13	Describe the methods of handling and summarizing the data that were charted.	16
**RESULTS**			
Selection of sources of evidence	14	Give numbers of sources of evidence screened, assessed for eligibility, and included in the review, with reasons for exclusions at each stage, ideally using a flow diagram.	4, Figure 1
Characteristics of sources of evidence	15	For each source of evidence, present characteristics for which data were charted and provide the citations.	Supplementary Information – Table 2
Critical appraisal within sources of evidence	16	If done, present data on critical appraisal of included sources of evidence (see item 12).	Categorization of Levels of Evidence – Table 4
Results of individual sources of evidence	17	For each included source of evidence, present the relevant data that were charted that relate to the review questions and objectives.	Supplementary Information – Table 2
Synthesis of results	18	Summarize and/or present the charting results as they relate to the review questions and objectives.	4-10, Figures 1-5, Table 1-3
**DISCUSSION**			
Summary of evidence	19	Summarize the main results (including an overview of concepts, themes, and types of evidence available), link to the review questions and objectives, and consider the relevance to key groups.	10-12
Limitations	20	Discuss the limitations of the scoping review process.	12
Conclusions	21	Provide a general interpretation of the results with respect to the review questions and objectives, as well as potential implications and/or next steps.	13
**FUNDING**			
Funding	22	Describe sources of funding for the included sources of evidence, as well as sources of funding for the scoping review. Describe the role of the funders of the scoping review.	16

JBI = Joanna Briggs Institute; PRISMA-ScR = Preferred Reporting Items for Systematic reviews and Meta-Analyses extension for Scoping Reviews.

* Where *sources of evidence* (see second footnote) are compiled from, such as bibliographic databases, social media platforms, and Web sites.

† A more inclusive/heterogeneous term used to account for the different types of evidence or data sources (e.g., quantitative and/or qualitative research, expert opinion, and policy documents) that may be eligible in a scoping review as opposed to only studies. This is not to be confused with *information sources* (see first footnote).

‡ The frameworks by Arksey and O'Malley (6) and Levac and colleagues (7) and the JBI guidance (4, 5) refer to the process of data extraction in a scoping review as data charting.

§ The process of systematically examining research evidence to assess its validity, results, and relevance before using it to inform a decision. This term is used for items 12 and 19 instead of "risk of bias" (which is more applicable to systematic reviews of interventions) to include and acknowledge the various sources of evidence that may be used in a scoping review (e.g., quantitative and/or qualitative research, expert opinion, and policy document).

Supplementary Information Table 2: Overview of full Study Details, Application Details and Data Details for all included studies

Study Details						Application Details						Data Details										
**Study Title**	**Study Author**	**DOI**	**Year of Publication**	**Journal**	**Country of Participants**	**mHealth Category**	**Medical Device Status**	**Medical Device Status_ Information Available**	**Application Name**	**Medical Specialty**	**Intended App Purpose**	**Data Collection Mode**	**Data Category**	**Data Type**	**In-App-Survey_ Validated Instrument**	**Data Collection Frequency**	**Data Collection Frequency_ Details**	**Parameter 1**	**Parameter 1_ Measurement Tool**	**Parameter 2**	**Parameter 2_ Measurement Tool**	**Study Endpoints**
Real-World Analysis of Remote Electrical Neuromodulation (REN) for the Acute Treatment of Migraine	Ailani et al.	10.3389/fpain.2021.753736	2022	FRONTIERS IN PAIN RESEARCH	United States	Treat specific condition	Medical Device	Yes	REN device (Nerivio®)	Nervous System	Pain Reduction	Active	User Input	In-App-Survey	No	Continuous	Daily	Pain Level	Own set of questions; Medication use	Treatment Intensity and Functional Disability	Output of the stimulator, as determined by the patient via the application	Pain & Physiological function
Evaluation of a Commercial Mobile Health App for Depression and Anxiety (AbleTo Digital+): Retrospective Cohort Study.	Anton et al.	10.2196/27570	2021	JMIR FORMATIVE RESEARCH	United States	Treat specific condition	Medical Device	No	AbleTo Digital+	Mental Health	Symptom Improvement	Active	User Input	In-App-Survey	Yes	Continuous	At the end of each module	Anxiety severity	Generalised Anxiety Disorder-7 (GAD-7); Anxiety Severity (SPIN)	Depression severity	Patient Health Questionnaire-9 (PHQ-9)	Depressive Symptoms & Anxiety Severity
Association Between Improvement in Baseline Mood and Long-Term Use of a Mindfulness and Meditation App: Observational Study.	Athanas et al.	10.2196/12617	2019	JMIR MENTAL HEALTH	Multiple:	Promoting good health	Wellness	No	Stop, Breathe & Think (SBT) app	General Wellbeing	Maintain wellbeing	Active	User Input	In-App-Survey	No	Continuous	Before and after meditation	Emotional State	"Emotional check-in" with 115 emotions to choose from			Emotional State
Digital Care for Chronic Musculoskeletal Pain: 10,000 Participant Longitudinal Cohort Study.	Bailey et al.	10.2196/18250	2020	JOURNAL OF MEDICAL INTERNET RESEARCH	United States	Treat specific condition	Medical Device	No	Hinge Health app	Musculoskeletal System	Pain Reduction	Active	User Input	In-App-Survey	yes	Periodic	Weekly plus unprompted	Pain Level	Visual analog scale (VAS)	App Engagement	Number of Sessions	Pain
Weight Loss Following Use of a Smartphone Food Photo Feature: Retrospective Cohort Study.	Ben Neriah et al.	10.2196/11917	2019	JMIR MHEALTH AND UHEALTH	United States	Health and Care Diaries	Wellness	Yes	Lose It! by FitNow	Endocrine and Metabolic System	Weight loss	Active	User Input	Vital Stats	n/a	Intermittent	time points self-selected	Body weight	Body weight measurement			Body weight
Perfect-use and typical-use Pearl Index of a contraceptive mobile app.	Berglund Scherwitzl et al.	10.1016/j.contraception.2017.08.014	2017	Contraception	Unclear	Drive clinical management	Medical Device	Yes	Natural Cycles	Reproductive health	Fertility management	Active	User Input	Event tags	n/a	Continuous	Daily at best	Secual acitivity, Pregnancy and Mode of pregnancy planning	n/a			Unintended pregnancies (Pearl Index)
Relationships Between Blood Pressure Reduction, Weight Loss, and Engagement in a Digital App-Based Hypertension Care Program: Observational Study.	Branch et al.	10.2196/38215	2022	JMIR FORMATIVE RESEARCH	United States	Inform clinical management	Medical Device	No	Lark Hypertension Care Program app	Cardiovascular System	Blood pressure management	Passive or Active	User Input, Device generated	Vital Stats or Connected Device	n/a	Continuous	Daily at best	Blood pressure	n/a			Blood pressure
Typical use effectiveness of Natural Cycles: postmarket surveillance study investigating the impact of previous contraceptive choice on the risk of unintended pregnancy.	Bull et al.	10.1136/bmjopen-2018-026474	2019	BMJ	Sweden	Drive clinical management	Medical Device	Yes	Natural Cycles	Reproductive health	Fertility management	Active	User Input	Event tags	n/a	Continuous	Daily at best	Secual acitivity, Pregnancy and Mode of pregnancy planning	n/a			Unintended pregnancies (Pearl Index)
The Relationship Between Weight Loss Outcomes and Engagement in a Mobile Behavioral Change Intervention: Retrospective Analysis	Carey et al.	10.2196/30622	2021	JMIR MHEALTH AND UHEALTH	Multiple:	Promoting good health	Wellness	Yes	Noom Coach app	Endocrine and Metabolic System	Weight loss	Active	User Input	Vital Stats	n/a	Intermittent	time points self-selected	Body weight	Body weight measurement			Body weight
Seeing the "Big" Picture: Big Data Methods for Exploring Relationships Between Usage, Language, and Outcome in Internet Intervention Data.	Carpenter et al.	10.2196/jmir.5725	2016	JOURNAL OF MEDICAL INTERNET RESEARCH	United States	Promoting good health	Wellness	No	Happify, now Ensemble	Mental Health	Maintain wellbeing	Active	User Input	In-App-Survey	No	Intermittent	At different time points after use	Positive Emotion	Happify Scale	Satisfaction with life	Happify Scale	Well-being

The Effect of an Automated Mobile Patient Engagement Application on Emergency Department Revisits: Prospective Observational Study.	Chatterjee et al.	10.2196/17839	2021	JMIR FORMATIVE RESEARCH	United States	Drive clinical management	Medical Device	No	GetWell Loop	Cardiovascular System	Avoid Readmission to Hospital	Passive	System Generated	Admission/Discharge and Progress Reports (EHR)	n/a	One-off	At beginning of use	ED Admissions	30-day revisit			Reduction of emergency department visits
Successful weight reduction and maintenance by using a smartphone application in those with overweight and obesity.	Chin et al.	10.1038/srep34563	2016	Scientific Reports (Nature)	Unclear	Promoting good health	Wellness	Yes	Noom Coach app	Endocrine and Metabolic System	Weight loss	Active	User Input	Vital Stats	n/a	Intermittent	time points self-selected	Body weight	Body weight measurement			Body weight
A Mobile-Based Nutrition Tracker App Enhanced Dietitian-Guided 2:1:1 Diet-Induced Weight Loss: An 8-Week Retrospective Cohort Study in Taiwan.	Chueh et al.	10.3390/nu16142331	2024	nutrients	Taiwan	Promoting good health	Wellness	No	COFIT application	Endocrine and Metabolic System	Weight loss	Active	User Input	Vital Stats	n/a	Intermittent	time points self-selected	Body weight	Body weight measurement			Body weight
Implementing Systematically Collected User Feedback to Increase User Retention in a Mobile App for Self-Management of Low Back Pain: Retrospective Cohort Study.	Clement et al.	10.2196/10422	2018	JMIR MHEALTH AND UHEALTH	Germany	Treat specific condition	Medical Device	Yes	Kaia	Musculoskeletal System	Pain Reduction	Active	User Input	In-App-Survey	yes	Continuous	Daily	Pain Level	Numeric Rating Scale (NRS)			Pain
Dosage Frequency Effects on Treatment Outcomes Following Self-managed Digital Therapy: Retrospective Cohort Study.	Cordella et al.	10.2196/36135	2022	JOURNAL OF MEDICAL INTERNET RESEARCH	United States	Treat specific condition	Medical Device	No	Constant Therapy	Cardiovascular System	Speech, Language, Cognitive Rehabilitation	Passive	Device Generated	App Engagement	n/a	Continuous	All the time	App Engagement	Time stamps; Item completion indicators; progression order			Domain scores (increase in difficulty level as percentage of total activities)
Engagement in an mHealth-Guided Exercise Therapy Program Is Associated With Reductions in Chronic Musculoskeletal Pain.	Delgado et al.	10.1097/PHM.0000000000002257	2023	American Journal of Physical Medicine & Rehabilitation	United States	Treat specific condition	Medical Device	No	SimpleTherapy	Musculoskeletal System	Pain Reduction	Active	User Input	In-App-Survey	yes	Continuous	At the beginning of each module	Pain Level	Numeric Rating Scale (NRS)			Pain
Olfactory Training and Visual Stimulation Assisted by a Web Application for Patients With Persistent Olfactory Dysfunction After SARS-CoV-2 Infection: Observational Study.	Denis et al.	10.2196/29583	2021	JOURNAL OF MEDICAL INTERNET RESEARCH	France	Treat specific condition	Medical Device	No	covidanosmia.eu web application	Nervous System	Symptom Improvement	Active	User Input	In-App-Survey	No	Continuous	At the end of each module	Olfactory ability	Visual analog scale (VAS)			Intensity of Olfactory Dysfunction
A Suicide Prevention Digital Technology for Individuals Experiencing an Acute Suicide Crisis in Emergency Departments: Naturalistic Observational Study of Real-World Acceptability, Feasibility, and Safety.	Dimeff et al.	10.2196/52293	2024	JMIR FORMATIVE RESEARCH	United States	Drive clinical management	Medical Device	No	Jaspr Health	Mental Health	Symptom Improvement	Active	User Input	In-App-Survey	No	Intermittent	time points self-selected	Adverse Events (AEs)	n/a	Emotional State	Items from the Safety and Imminent Distress Questionnaire; Rating of feelings on a numerical scale	Agitation and Distress & Number of Adverse Events
Digital health program improves quality of life in rheumatoid arthritis: a retrospective analysis of real-world data.	Dobies et al.	10.55563/clinexprheumatol/mg5n9	2024	Clinical and Experimental Rheumatology	Finland	Treat specific condition	Medical Device	Yes	Sidekick Health RA programme	Cardiovascular System	Symptom Improvement	Active	User Input	In-App-Survey	No	Continuous	At time of use	Symptom Severity	Quality of life likert scale			Quality of Life
The Efficacy of an mHealth App in Facilitating Weight Loss Among Japanese Fitness Center Members: Regression Analysis Study.	Eguchi et al.	10.2196/48435	2023	JMIR FORMATIVE RESEARCH	Japan	Health and Care Diaries	Wellness	No	Calomama Plus	General Wellbeing	Weight loss	Active	User Input	Vital Stats	n/a	Intermittent	time points self-selected	Body weight	n/a			Body weight

Effect of the smartphone application on caesarean section in women with overweight and obesity: a randomized controlled trial in China.	Feng et al.	10.1186/s12884-023-06004-7	2023	BMC Pregnancy and Childbirth	China	Treat specific condition	Medical Device	No	smartphone based weight management application (App)	Endocrine and Metabolic System	Avoid Complication	Passive	System Generated	Procedures (EHR)	n/a	One-off	At time of use	Caesarean section				Number of caesarean sections
Role of Digital Engagement in Diabetes Care Beyond Measurement: Retrospective Cohort Study.	Fundoiano-Hershcovitz et al.	10.2196/24030	2021	JMIR DIABETES	Israel	Treat specific condition	Medical Device	No	Dario digital therapeutics solution	Endocrine and Metabolic System	Disease Management	Passive	Device Generated	Connected Device	n/a	Continuous	At time of use	Blood glucose level	from connected device			Blood glucose level
Real-World Evidence of Improved Glycemic Control in People with Diabetes Using a Bluetooth-Connected Blood Glucose Meter with a Mobile Diabetes Management App.	Grady et al.	10.1089/dia.2022.0134	2022	DIABETES TECHNOLOGY & THERAPEUTICS	Multiple:	Inform clinical management	Medical Device	No	OneTouch Reveal (OTR) mobile app	Endocrine and Metabolic System	Disease Management	Passive	Device Generated	Connected Device	n/a	Continuous	At time of use	Blood glucose level	from connected device			Blood glucose level
Sustained Improvements in Readings in-Range Using an Advanced Bluetooth Connected Blood Glucose Meter and a Mobile Diabetes App: Real-World Evidence from more than 55,000 People with Diabetes.	Grady et al.	10.1007/s13300-023-01415-3	2023	Diabetes Therapy	United Kingdom	Inform clinical management	Medical Device	No	OneTouch Reveal (OTR) mobile app	Endocrine and Metabolic System	Disease Management	Passive	Device Generated	Connected Device	n/a	Continuous	At time of use	Blood glucose level	from conncected device			Blood glucose level
Internet-based cognitive behavioural therapy in the real world: Naturalistic use and effectiveness of an evidence-based platform in New Zealand.	Guiney et al.	10.1177/00048674231183641	2024	Australian & New Zealand Journal of Psychiatry	New Zealand	Treat specific condition	Medical Device	No	Just a thought	Mental Health	Symptom Improvement	Active	User Input	In-App-Survey	Yes	Continuous	Before each module	Symptom Severity	Kessler psychological distress scale-10 (K10)			Psychological Distress
Real-World Evidence of Aripiprazole Tablets with Sensor: Treatment Patterns and Impacts on Psychiatric Healthcare Resource Utilization.	Hadzi Boskovic et al.	10.2147/CEOR.S402357	2023	ClinicoEconomics and Outcomes Research	United States	Inform clinical management	Medical Device	No	Aripiprazole tablets with sensor	Mental Health	Adherence improvement	Passive	System Generated	Medication Use (claims), Admission/Discharge and Progress Reports (claims)	n/a	One-off		ED Admissions		Medication use		Psychiatric pharmacy claims & Inpatient and outpatient admissions & psychiatric ED claims
Real-World Evidence From a Digital Health Treatment Program for Female Urinary Incontinence: Observational Study of Outcomes Following User-Centered Product Design.	Hall et al.	10.2196/58551	2024	JMIR FORMATIVE RESEARCH	United States	Treat specific condition	Medical Device	No	Leva Pelvic Health System	Genitourinary System	Symptom Improvement	Active	User Input	In-App-Survey	Yes	One-off	Baseline and after 4 weeks	Symptom Severity Questionnaire	Urogenital Distress Inventory Short Form (UDI-6)			Urogenital Distress
Implementing digital mental health interventions at scale: one-year evaluation of a national digital CBT service in Ireland.	Harty et al.	10.1186/s13033-023-00592-9	2023	International Journal of Mental Health Systems	Ireland	Treat specific condition	Medical Device	No	Space from Depression, Space from Anxiety, Space from Depression and Anxiety, and Space from Generalised Anxiety Disorder by SilverCloud	Mental Health	Symptom Improvement	Active	User Input	In-App-Survey	Yes	Periodic	bi-weekly basis	Symptom Severity Questionnaire	Patient Health Questionnaire-9 (PHQ-9)	Symptom Severity Questionnaire	Generalised Anxiety Disorder-7 (GAD-7); Work and Social Adjustment Scale (WSAS)	Depressive Symptoms & Anxiety Severity & Work and Social Adjustment
Digital hypertension management: clinical and cost outcomes of a pilot implementation of the OMRON hypertension management platform.	Holmstrand et al.	10.3389/fdgth.2023.1128553	2023	Frontiers in Digital Health	United States	Inform clinical management	Medical Device	No	Omron	Cardiovascular System	Blood pressure management	Passive	Device Generated	Connected Device	n/a	Continuous	At time of use	Blood pressure	Sensor			Blood pressure
The effectiveness of mobile app usage in facilitating weight loss: An observational study.	Huntriss et al.	10.1002/osp4.757	2024	Obesity Science & Practice	Unclear	Promoting good health	Wellness	No	SIMPLE mobile application	Endocrine and Metabolic System	Weight loss	Active	User Input	Vital Stats	n/a	Intermittent	During assessment period	Body weight	n/a			Body weight

Effectiveness of a Digital Lifestyle Change Program in Obese and Type 2 Diabetes Populations: Service Evaluation of Real-World Data.	Idris et al.	10.2196/15189	2020	JMIR DIABETES	United Kingdom	Promoting good health	Wellness	No	OurPath / Second Nature	Endocrine and Metabolic System	Weight loss	Passive	Device Generated	Connected Device	n/a	Continuous	At time of use	Body weight	Wireless scale			Body weight
An Empathy-Driven, Conversational Artificial Intelligence Agent (Wysa) for Digital Mental Well-Being: Real-World Data Evaluation Mixed-Methods Study.	Inkster et al.	10.2196/12106	2018	JMIR MHEALTH AND UHEALTH	Multiple:	Treat specific condition	Medical Device	Yes	Wysa App	Mental Health	Symptom Improvement	Active	User Input	In-App-Survey	Yes	Intermittent	At various points of app use	Depression severity	Patient Health Questionnaire-2 (PHQ-2)			Depressive Symptoms
Perfect- and typical-use effectiveness of the Dot fertility app over 13 cycles: results from a prospective contraceptive effectiveness trial.	Jennings et al.	10.1080/13625187.2019.1581164	2019	The European Journal of Contraception & Reproductive Health Care	United States	Drive clinical management	Medical Device	Yes	Dynamic Optimal Timing (Dot) / Clue	Reproductive health	Fertility management	Active	User Input	Event tags	n/a	Continuous	At time of use	Physiological parameters	Period start date	Secual acitivity, Pregnancy and Mode of pregnancy planning	n/a	Typical Use effectiveness & Perfect Use Effectiveness
The Diabits App for Smartphone-Assisted Predictive Monitoring of Glycemia in Patients With Diabetes: Retrospective Observational Study.	Kriventsov et al.	10.2196/18660	2020	JMIR DIABETES	Canada	Inform clinical management	Medical Device	No	Diabits	Endocrine and Metabolic System	Disease Management	Passive	Device Generated	Connected Device	n/a	Continuous	At time of use	Blood glucose level	Sensor	Time in euglycemic range	Sensor	Blood glucose level
Impact of a Combined Continuous Glucose Monitoring-Digital Health Solution on Glucose Metrics and Self-Management Behavior for Adults With Type 2 Diabetes: Real-World, Observational Study.	Kumbara et al.	10.2196/47638	2023	JMIR DIABETES	United States	Inform clinical management	Medical Device	Yes	The BlueStar (Welldoc)	Endocrine and Metabolic System	Disease Management	Passive	Device Generated	Connected Device	n/a	Continuous	At time of use	Blood glucose level	Sensor	Time in euglycemic range	Sensor	Blood glucose level
App Engagement as a Predictor of Weight Loss in Blended-Care Interventions: Retrospective Observational Study Using Large-Scale Real-World Data.	Lehmann et al.	10.2196/45469	2024	JOURNAL OF MEDICAL INTERNET RESEARCH	Multiple:	Inform clinical management	Medical Device	Yes	Oviva	Endocrine and Metabolic System	Weight loss	Active	User Input	Vital Stats	n/a	Intermittent	During assessment period	Body weight	n/a			Body weight
Digital cognitive behavior therapy for insomnia improving sleep quality: a real-world study.	Liang et al.	10.1186/s12888-022-04411-2	2022	BMC Psychiatry	China	Treat specific condition	Medical Device	No	Good Sleep 365 Days	Mental Health	Symptom Improvement	Active	User Input	In-App-Survey	Yes	Intermittent	During assessment period	Sleep Quality	Pittsburgh Sleep Quality Index (PSQI)			Sleep Quality
Dose-response relationship between computerized cognitive training and cognitive improvement.	Liu et al.	10.1038/s41746-024-01210-9	2024	npj | digital medicine	China	Treat specific condition	Medical Device	No	CCT platform	Mental Health	Symptom Improvement	Passive	Device Generated	Task Performance	n/a	Continuous	At time of use	Cognitive ability	Performance in tasks	App Engagement	Trainings per day; Minutes of training	Cognitive Index
Comparative Effectiveness of Digital Cognitive Behavioral Therapy vs Medication Therapy Among Patients With Insomnia.	Lu et al.	10.1001/jamanetworkopen.2023.7597	2023	JAMA Network Open	China	Treat specific condition	Medical Device	No	Good Sleep 365 Days	Mental Health	Symptom Improvement	Active	User Input	In-App-Survey	Yes	Intermittent	During assessment period	Sleep Quality	Pittsburgh Sleep Quality Index (PSQI)			Sleep Quality
A Personalized Approach Bias Modification Smartphone App ("SWiPE") to Reduce Alcohol Use: Open-Label Feasibility, Acceptability, and Preliminary Effectiveness Study.	Manning et al.	10.2196/31353	2021	JMIR MHEALTH AND UHEALTH	Australia	Promoting good health	Wellness	No	SWiPE	Mental Health	Symptom Improvement	Active	User Input	In-App-Survey	No	Intermittent	During assessment period	Alcohol Consumption	Frequency and quantity	Alcohol Craving	Craving Experience Questionnaire (CEQ-F)	Alcohol Consumption & Alcohol Craving
Guided Internet-Based Cognitive Behavioral Therapy for Adult Depression and Anxiety in Routine Secondary Care: Observational Study.	Mathiasen et al.	10.2196/10927	2018	JOURNAL OF MEDICAL INTERNET RESEARCH	Denmark	Treat specific condition	Medical Device	No	Internetpsykiatrien	Mental Health	Symptom Improvement	Active	User Input	In-App-Survey	Yes	Periodic	Weekly	Depression severity	Patient Health Questionnaire-9 (PHQ-9)	Anxiety severity	Generalised Anxiety Disorder-7 (GAD-7)	Depressive Symptoms & Anxiety Severity

The Impact of a Gamified Intervention on Daily Steps in Real-Life Conditions: Retrospective Analysis of 4800 Individuals.	Mazéas et al.	10.2196/47116	2024	JOURNAL OF MEDICAL INTERNET RESEARCH	France	Health and Care Diaries	Wellness	No	Kiplin	General Wellbeing	Increase Physical Activity	Passive	Device Generated	Activity Data	n/a	Continuous	At time of use	Physical activity	Stepcount			Daily Step Count
Acceptability and Effectiveness of Artificial Intelligence Therapy for Anxiety and Depression (Youper): Longitudinal Observational Study.	Mehta et al.	10.2196/26771	2021	JOURNAL OF MEDICAL INTERNET RESEARCH	United States	Treat specific condition	Medical Device	No	Youper	Mental Health	Symptom Improvement	Active	User Input	In-App-Survey	No	Periodic	14 days	Anxiety severity	Generalised Anxiety Disorder-7 (GAD-7)	Depression severity	Patient Health Questionnaire-9 (PHQ-9)	Depressive Symptoms & Anxiety Severity
Weight Gain Prevention Outcomes From a Pragmatic Digital Health Intervention With Community Health Center Patients: Randomized Controlled Trial.	Miller et al.	10.2196/50330	2024	JOURNAL OF MEDICAL INTERNET RESEARCH	United States	Promoting good health	Wellness	No	Balance weight gain prevention intervention	Endocrine and Metabolic System	Weight loss	Passive	System Generated	Vital Stats (EHR)	n/a	Continuous	Not applicable	Body weight	At regular primary care visit			Body weight
Introducing and Evaluating the Effectiveness of Online Cognitive Behavior Therapy for Gambling Disorder in Routine Addiction Care: Comparative Cohort Study.	Molander et al.	10.2196/54754	2024	JOURNAL OF MEDICAL INTERNET RESEARCH	Sweden	Treat specific condition	Medical Device	No	iCBT program	Mental Health	Symptom Improvement	Active	User Input	In-App-Survey	Yes	Continuous	Before each module	Symptom Severity	Gambling Symptom Assessment Scale (GSAS)	Gambling Activity	Yes/No	Gambling Symptoms & Gambling Activity
The Effects of a Digital Mental Health Intervention in Adults With Cardiovascular Disease Risk Factors: Analysis of Real-World User Data.	Montgomery et al.	10.2196/32351	2021	JMIR CARDIO	United States	Promoting good health	Wellness	No	Happify, now Ensemble	Mental Health	Symptom Improvement	Active	User Input	In-App-Survey	Yes	Periodic	Every 2 weeks	Symptom Severity	Generalised Anxiety Disorder-2 (GAD-2)	Well-being	Happify Scale	Well-being & Anxiety Severity
The Effectiveness of an App (Insulia) in Recommending Basal Insulin Doses for French Patients With Type 2 Diabetes Mellitus: Longitudinal Observational Study.	Nevoret et al.	10.2196/44277	2023	JMIR DIABETES	France	Drive clinical management	Medical Device	No	Insulia	Endocrine and Metabolic System	Disease Management	Active	User Input	Vital Stats	n/a	Intermittent	At various points of app use	Blood glucose level	Achievement of target fasting blood glucose (FBG) level			Blood glucose level
The Effects of a Digital Well-Being Intervention on Patients With Chronic Conditions: Observational Study.	Parks et al.	10.2196/16211	2020	JOURNAL OF MEDICAL INTERNET RESEARCH	United States	Promoting good health	Wellness	No	Happify, now Ensemble	Mental Health	Symptom Improvement	Active	User Input	In-App-Survey	No	Periodic	Every 2 weeks	Well-being	Happify Scale			Well-being
Validation of COPDPredict™: Unique Combination of Remote Monitoring and Exacerbation Prediction to Support Preventative Management of COPD Exacerbations.	Patel et al.	10.2147/COPD.S309372	2021	International Journal of Chronic Obstructive Pulmonary Disease	United Kingdom	Drive clinical management	Medical Device	Yes	COPDPredict	Respiratory System	Monitoring physiological parameters	Passive and Active	User Input, Device generated	In-App-Survey & Connected Device	No	Continuous	At time of use	Well-being	Five-Level Likert Score	Physiological parameters	Lung function	Well-being & lung function
Natural Cycles app: contraceptive outcomes and demographic analysis of UK users.	Pearson et al.	10.1080/13625187.2020.1867844	2021	The European Journal of Contraception & Reproductive Health Care	United Kingdom	Drive clinical management	Medical Device	Yes	Natural Cycles	Reproductive health	Fertility management	Active	User Input	Event tags	n/a	Continuous	Daily at best	Secual acitivity, Pregnancy and Mode of pregnancy planning	n/a			Unintended pregnancies (Pearl Index)
Home blood pressure measurement self-reporting in real-life practices using the Hy-Result app: self-monitoring and digital pathway.	Postel-Vinay et al.	10.21037/mhealth-23-66	2024	mHealth	France	Inform clinical management	Medical Device	No	Hy-Result	Cardiovascular System	Blood pressure management	Active	User Input	Vital Stats	n/a	Continuous	Every day	Blood pressure	Manual Input			Blood pressure
Determining the Evolution of Headache Among Regular Users of a Daily Electronic Diary via a Smartphone App: Observational Study.	Raffaelli et al.	10.2196/26401	2021	JMIR MHEALTH AND UHEALTH	Germany	Inform clinical management	Medical Device	Yes	M-sense	Nervous System	Symptom Improvement	Active	User Input	In-App-Survey	No	Intermittent	At optional time points	Headache information	Number of Monthly Headache Days; Monthly Migraine Days	Medication use	acute headache medication	Headache and migraine frequency & intensity

The Potential of a Digital Weight Management Program to Support Specialist Weight Management Services in the UK National Health Service: Retrospective Analysis.	Richards et al.	10.2196/52987	2024	JMIR DIABETES	United Kingdom	Promoting good health	Wellness	No	OurPath / Second Nature	Endocrine and Metabolic System	Weight loss	Passive	Device Generated	Connected Device	n/a	Periodic	Yearly	Body weight	At baseline, 1 and 2 years			Body weight
Real-world evidence from users of a behavioral digital therapeutic for chronic insomnia.	Ritterband et al.	10.1016/j.brat.2022.104084	2022	Behaviour Research and Therapy	United States	Treat specific condition	Medical Device	No	SHUTi (Sleep Health Using the Internet)	Mental Health	Symptom Improvement	Active	User Input	In-App-Survey	Yes	Continuous	Daily at best	Insomnia severity	Insomnia Severity Index (ISI)	Sleep Quality	Sleep efficiency, Sleep Onset Latency (SOL), Wake after sleep onset (WASO), early morning awakening (EMA)	Sleep Quality & Insomnia severity
Effects of app-based relaxation techniques on perceived momentary relaxation: Observational data analysis in people with cancer.	Schläpfer et al.	10.1016/j.jpsychores.2024.111864	2024	Journal of Psychosomatic Research	Switzerland	Promoting good health	Wellness	No	CanRelax app 2.0	Mental Health	Symptom Improvement	Active	User Input	In-App-Survey	No	Periodic	At every third core	Relaxation	Ecological momentary assessments (EMA)			Relaxation
A Fully Automated Conversational Artificial Intelligence for Weight Loss: Longitudinal Observational Study Among Overweight and Obese Adults.	Stein et al.	10.2196/diabetes.8590	2017	JMIR DIABETES	United States	Inform clinical management	Medical Device	No	Lark Weight Loss Health Coach AI (HCAI)	Endocrine and Metabolic System	Weight loss	Passive	Device Generated	Connected Device	n/a	One-off	Baseline and after 4 weeks	Body weight				Body weight
Postmarketing Follow-Up of a Digital Home Exercise Program for Back, Hip, and Knee Pain: Retrospective Observational Study With a Time-Series and Matched-Pair Analysis.	Teepe et al.	10.2196/43775	2023	JOURNAL OF MEDICAL INTERNET RESEARCH	Germany	Treat specific condition	Medical Device	Yes	Vivira	Musculoskeletal System	Pain Reduction	Active	User Input	In-App-Survey	Yes	Periodic	Weekly	Pain Level	Verbal-numerical rating scale (VNRS)	Motor function	Self reported assessment of functional state	Pain & Physiological function
Preliminary Use and Outcome Data of a Digital Home Exercise Program for Back, Hip, and Knee Pain: Retrospective Observational Study With a Time Series and Matched Analysis.	Teepe et al.	10.2196/38649	2022	JMIR MHEALTH AND UHEALTH	Germany	Treat specific condition	Medical Device	Yes	Vivira	Musculoskeletal System	Pain Reduction	Active	User Input	In-App-Survey	Yes	Periodic	Every 2 weeks	Pain Level	Verbal-numerical rating scale (VNRS)			Pain
Effect of a prescription digital therapeutic for chronic insomnia on post-treatment insomnia severity, depression, and anxiety symptoms: results from the real-world DREAM study.	Thorndike et al.	10.3389/fpsyt.2024.1450615	2024	Frontiers in Psychiatry	United States	Treat specific condition	Medical Device	No	PDT; Somryst	Mental Health	Symptom Improvement	Active	User Input	In-App-Survey	Yes	Continuous	Before each module	Insomnia severity	Insomnia Severity Index (ISI)	Symptom Severity Questionnaire	Patient Health Questionnaire (PHQ-8); Generalized Anxiety Disorder-7 scale (GAD-7)	Insomnia Severity & Depressive Symptoms & Anxiety Severity
The Effects of Continuous Usage of a Diabetes Management App on Glycemic Control in Real-world Clinical Practice: Retrospective Analysis.	Tu et al.	10.2196/23227	2021	JOURNAL OF MEDICAL INTERNET RESEARCH	Taiwan	Inform clinical management	Medical Device	No	Health2Sync	Endocrine and Metabolic System	Disease Management	Active	User Input	Vital Stats	n/a	Intermittent	During assessment period	Blood glucose level	HbA1 records			Blood glucose level
Personalised Nutritional Recommendations Based on Individual Post-Prandial Glycaemic Responses Improve Glycaemic Metrics and PROMs in Patients with Type 2 Diabetes: A Real-World Assessment.	Ungersboeck et al.	10.3390/nu14102123	2022	nutrients	Germany	Inform clinical management	Medical Device	Yes	Una health app	Endocrine and Metabolic System	Disease Management	Passive	Device Generated	Connected Device	n/a	Continuous	Daily at best	Blood glucose level				Blood glucose level

"Back Rx, a personalized mobile phone application for discogenic chronic low back pain: a prospective pilot study"	Vad et al.	10.1186/s12891-022-05883-9	2022	BMC Musculoskeletal Disorders	United States	Treat specific condition	Medical Device	No	Back Rx	Musculoskeletal System	Pain Reduction	Active	User Input	In-App-Survey	yes	Periodic	3 weeks, 6 weeks and 3 month	Pain Level	Visual Analog Scale (VAS)	Functional Disability	Your Activities of Daily Living (YADL)	Pain
Changes in Perceived Stress Following a 10-Week Digital Mindfulness-Based Stress Reduction Program: Retrospective Study.	Venkatesan et al.	10.2196/25078	2021	JMIR FORMATIVE RESEARCH	United States	Treat specific condition	Medical Device	No	Vida health app	Mental Health	Symptom Improvement	Active	User Input	In-App-Survey	Yes	Periodic	Every 2 weeks	Perceived Stress	Perceived Stress Scale (PSS-10)			Stress
Digital Cognitive Behavior Therapy Intervention for Depression and Anxiety: Retrospective Study.	Venkatesan et al.	10.2196/21304	2020	JMIR MENTAL HEALTH	United States	Treat specific condition	Medical Device	No	Vida health app	Mental Health	Symptom Improvement	Active	User Input	In-App-Survey	Yes	Periodic	Every 2 weeks	Depressive Symptoms	Patient Health Questionnaire (PHQ-8)	Anxiety severity	Generalized Anxiety Disorder-7 scale (GAD-7)	Depressive Symptoms & Anxiety Severity
Improvements in Depression Outcomes Following a Digital Cognitive Behavioral Therapy Intervention in a Polychronic Population: Retrospective Study.	Venkatesan et al.	10.2196/38005	2022	JMIR FORMATIVE RESEARCH	United States	Treat specific condition	Medical Device	No	Vida health app	Mental Health	Symptom Improvement	Active	User Input	In-App-Survey	Yes	Periodic	Every 6 weeks	Depressive Symptoms	Patient Health Questionnaire (PHQ-8)	Anxiety severity	Generalized Anxiety Disorder-7 scale (GAD-7)	Depressive Symptoms & Anxiety Severity
Mobile Sensing and Support for People With Depression: A Pilot Trial in the Wild.	Wahle et al.	10.2196/mhealth.5960	2016	JMIR MHEALTH AND UHEALTH	Switzerland	Treat specific condition	Medical Device	No	Mobile Sensing and Support (MOSS)	Mental Health	Symptom Improvement	Active	User Input	In-App-Survey	Yes	Periodic	Every 2 weeks	Depressive Symptoms	Patient Health Questionnaire (PHQ-9)			Depressive Symptoms & Relation between adherence and change
Opioid Initiation Within One Year After Starting a Digital Musculoskeletal (MSK) Program: An Observational, Longitudinal Study with Comparison Group.	Wang et al.	10.2147/JPR.S412081	2023	Journal of Pain Research	United States	Treat specific condition	Medical Device	No	Digital MSK Program	Musculoskeletal System	Pain Reduction	Passive	System Generated	Medication Use (claims data)	n/a	One-off	n/a	Medication use				Opioid use
App-Based Lifestyle Intervention (PINK! Coach) in Breast Cancer Patients-A Real-World-Data Analysis.	Wolff et al.	10.3390/cancers16051020	2024	cancers	Germany	Inform clinical management	Medical Device	Yes	PINK! Coach	Cancer	Disease Management	Passive and Active	User Input, Device generated	Vital Stats & Activity Data	n/a	Intermittent	During assessment period	Physical activity	Steps per day, activity minutes per day	Body weight	Self-reported	Physical activity & Body weight
Long-Term Results of a Digital Hypertension Self-Management Program: Retrospective Cohort Study.	Wu et al.	10.2196/43489	2023	JMIR CARDIO	United States	Inform clinical management	Medical Device	No	Omada for Hypertension	Cardiovascular System	Blood pressure management	Passive	Device Generated	Connected Device	n/a	Periodic	Monthly	Blood pressure	Sensor	Body weight		Blood pressure
Preliminary effectiveness of an evidence-based mobile application to promote resilience among working adults in Singapore and Hong Kong: Intensive longitudinal study.	YangToh et al.	10.1177/20552076231178616	2023	Digital Health	Multiple:	Promoting good health	Wellness	No	Intellect	Mental Health	Symptom Improvement	Active	User Input	In-App-Survey	No	Continuous	Daily at best	Mood Level	In app slider	Resilience Score	Four item slider	Resilience & mood
Effectiveness of Lilly Connected Care Program (LCCP) App-Based Diabetes Education for Patients With Type 2 Diabetes Treated With Insulin: Retrospective Real-World Study.	Zhang et al.	10.2196/17455	2020	JMIR MHEALTH AND UHEALTH	China	Inform clinical management	Medical Device	No	Lilly Connected Care Program (LCCP)	Endocrine and Metabolic System	Disease Management	Passive	Device Generated	Connected Device	n/a	One-off	Baseline and 12 weeks	Blood glucose level	Fasting Blood Glucose, Postprandial Blood glucose			Blood glucose level
Transitioning from Self-Monitoring of Blood Glucose to Continuous Glucose Monitoring in Combination with a mHealth App Improves Glycemic Control in People with Type 1 and Type 2 Diabetes.	Zivkovic et al.	10.1089/dia.2024.0169	2024	DIABETES TECHNOLOGY & THERAPEUTICS	Multiple:	Inform clinical management	Medical Device	Yes	mySugr	Endocrine and Metabolic System	Disease Management	Passive	Device Generated	Connected Device	n/a	Continuous	All the time	Blood glucose level	Sensor			Blood glucose level

Supplementary Information Table 3: Overview of full Study Design Details including Evidence Levels (adapted OCEBM/FDA RWE) and Key Sample Descriptors for all included studies

Study Details		Application Details		Data Details			Study Design Details											Comments
**DOI**	**Study Author**	**Application Name**	**Intended App Purpose**	**Measured Health Outcome (Study Endpoint)**	**RWD Category**	**RWD Parameter**	**Study Design_OCEBM / FDA RWE classification**	**Interventional vs. observational**	**Randomized Design**	**Temporal Orientation**	**Mechanisms of Comparison (How are the Outcomes compared?)**	**Details Comparison**	**Type of Comparator/Control (What is the intervention compared against?)**	**Details Comparator/Control**	**OCEBM / FDA Evidence Level**	**Number of Participants**	**Timeframe of Data Analysis [days]**	**Study Design Summary (How RWD is used in the study to demonstrate app effectiveness for intended purpose)**
10.3389/fpain.2021.753736	Ailani et al.	REN device (Nerivio®)	Pain Reduction	Pain & Physiological function	User Input	Pain Level<br><br>Treatment Intensity and Functional Disabilty	Pre-Post Single-Group Study	Observational	No	Retrospective	Intra-individual	n/a	No Direct Comparator	n/a	4	2.514	474	Longitudinal symptom tracking via the migraine diary are used in study for continuous RWD monitoring of health outcomes. Findings of pain reductions during app use are discussed in relation to previously published RCT results on REN's efficacy (active device vs. sham).
10.2196/27570	Anton et al.	AbleTo Digital+	Symptom Improvement	Depressive Symptoms & Anxiety Severity	User Input	Anxiety severity<br><br>Depression severity	Pre-Post Single-Group Study	Observational	No	Retrospective	Intra-individual & Inter-individual	Subgroups are based on program track, module completion, coaching use	No Direct Comparator	n/a	4	1.896	180	Longitudinal tracking of anxiety and depression symptoms through self-reported assessments with validated questionnaires (PHQ-9, GAD-7, SPIN), along with completion rates of CBT modules and participation in coaching sessions, are used in study for continuous RWD monitoring of health outcomes. Symptom reduction was analyzed by comparing inter-individual in groups who completed all modules versus those who discontinued use, showing greater improvements in high-completion users. General findings are described in relation to previous meta-analyses on CBT effectiveness in RCTs. Detailed comparisons (e.g., magnitude of score reduction) were not conducted.
10.2196/12617	Athanas et al.	Stop, Breathe & Think (SBT) app	Maintain wellbeing	Emotional State	User Input	Emotional State	Pre-Post Single-Group Study	Observational	No	Retrospective	Intra-individual	n/a	No Direct Comparator	n/a	4	120.000	180	Retrospective single-group pre-post cohort study evaluating the impact of the AbleTo Digital+ mobile app on symptoms of depression, generalized anxiety, and social anxiety among U.S. adults. Symptom scores (PHQ-9, GAD-7, or SPIN) were assessed at baseline and after each of up to eight cognitive behavioral therapy–based program modules, allowing for repeated intra-individual comparisons over time. Among users with at least mild baseline symptoms who completed four or more modules, large within-subject symptom reductions were observed across all programs.
10.2196/jmir.5725	Bailey et al.	Hinge Health app	Pain Reduction	Pain	User Input	Pain Level<br><br>App Engagement	Pre-Post Single-Group Study	Observational	No	Retrospective	Intra-individual & Inter-individual	Subgroups based on engagement or location of pain (posthoc)	No Direct Comparator	n/a	4	10.264	84	The study uses longitudinal RWD measured with validated questionnaire on pain level and app engagement. Pain reduction as health outcome was analyzed by intra- (baseline and end of study) and inter-individual comparison (program completers and non-completers). Findings of increased pain reduction correlated with higher app engagement. Findings were descriptively discussed in relation to existing literature on conservative care interventional studies.

10.2196/11917	Ben Neriah et al.	Lose It! by FitNow	Weight loss	Body weight	User Input	Body weight	Cohort Study	Observational	No	Retrospective	Intergroup	Cohorts based on different feature use	No Direct Comparator	n/a	3b	123.787	180	Retrospective cohort study examining the impact of a food photo feature in a mobile weight loss app (Lose It!) on weight loss outcomes. Adult users with overweight or obesity were grouped based on whether they used the optional photo-based food logging tool. The primary outcome was percent weight loss, calculated from self-reported weigh-ins. Weight and food intake were logged by users on a self-determined, event-driven basis. Weight loss outcomes are discussed for photo feature users and nonusers, forming a self-selected, non-randomized group.
10.1016/j.contraception.2017.08.014	Berglund Scherwitzl et al.	Natural Cycles	Fertility management	Unintended pregnancies (Pearl Index)	User Input	Secual acitivity, Pregnancy and Mode of pregnancy planning	Cohort Study	Observational	No	Prospective	Intergroup	Cohorts based an use behavior and data completeness	No Direct Comparator	n/a	3b	22.785	390	Prospective study uses user-reported or passively measured RWD to evaluate contraceptive effectiveness of app. Findings of contraception efficacy were discussed in relation to previous studies by same researcher and other digital contraception tools.
10.2196/38215	Branch et al.	Lark Hypertension Care Program app	Blood pressure management	Blood pressure	User Input, Device Generated	Blood pressure	Pre-Post Single-Group Study	Observational	No	Retrospective	Intra-individual & Inter-individual	Subgroups based on baseline BP	No Direct Comparator	n/a	4	2.861	180	Study uses longitudinal app collected blood pressure readings and weight to calculate change in blood pressure and weight as health outcome. The findings were compared begin and end of program within individuals and among program participants. The findings were discussed numerically in relation to literature on human-led behaviour interventions.
10.1136/bmjopen-2018-026474	Bull et al.	Natural Cycles	Fertility management	Unintended pregnancies (Pearl Index)	User Input	Secual acitivity, Pregnancy and Mode of pregnancy planning	Cohort Study	Observational	No	Prospective	Intergroup	Cohorts based on self-reported previous contraceptive method	No Direct Comparator	n/a	3b	16.331	365	Prospective observational cohort study evaluating the typical-use effectiveness of the Natural Cycles fertility awareness app among 16,331 women in Sweden. Participants self-selected into the study as paying users of the app for contraception and were categorized into cohorts based on their previously used contraceptive method (e.g., condoms, pills, hormonal IUDs). Daily data on temperature, sexual activity, and app usage were logged. The primary outcome was the 1-year Pearl Index (unintended pregnancies per 100 woman-years) for each cohort, and secondary outcomes included the 13-cycle failure rate and frequency of unprotected sex on fertile (red) days.
10.2196/30622	Carey et al.	Noom Coach app	Weight loss	Body weight	User Input	Body weight	Cohort Study	Observational	No	Retrospective	Intergroup	Cohorts based on weight changes (stable, moderate, high)	No Direct Comparator	n/a	3b	11.252	371	Retrospective study uses longitudinal RWD collected at self-selected timepoints to analyse body weight reduction. Findings were compared among different weight change groups (moderate loss and high loss against stable). Higher engagement levels were consistently correlated with higher weight reduction. Findings were descriptively discussed in relation to existing research on food-logging and weight loss.

10.2196/jmir.5725	Carpenter et al.	Happify, now Ensemble	Maintain wellbeing	Well-being	User Input	Positive Emoption<br><br>Satisfaction with life	Cohort Study	Observational	No	Retrospective	Intergroup & Intragroup	Cohorts based on app usage patterns and data availability	No Direct Comparator	n/a	3b	152.747	56	Retrospective study uses self-reported emotion data to evaluate symptom change within-person and across app engagement level groups. Findings of improved symptoms correlated with higher engagement. Findings were descriptively discussed in relation to existing evidence on CBT-based digital interventions.
10.2196/17839	Chatterjee et al.	GetWell Loop	Avoid Readmission to Hospital	Reduction of emergency department visits	System Generated	ED Admissions	Cohort Study	Observational	No	Prospective	Intergroup	n/a	Self-selected Comparator	Usual Care (Clinical Data)	2	3.866	365	Prospective cohort study evaluating the impact of a mobile patient engagement app (GetWell Loop) on 30-day emergency department revisits. Adult patients discharged home from two hospitals were invited to activate the app, which delivered automated postdischarge check-ins and self-care resources. Outcomes were compared between patients who activated the app and those who did not, forming a concurrent, non-randomized control group. The primary outcome was any ED revisit within 30 days of discharge, assessed via Maryland's statewide health information exchange. App activation and usage data were tracked through the digital platform.
10.1038/srep34563	Chin et al.	Noom Coach app	Weight loss	Body weight	User Input	Body weight	Cohort Study	Observational	No	Retrospective	Intergroup	Cohorts based on weight change trajectory	No Direct Comparator	n/a	3b	35.921	182	To prove effectiveness of app the study analysed user-logged weight data calculating weight change from baseline to end of program. Study identifies significant correlations between input frequency and weight loss success while controlling for confounders through multivariate regression. Findings of weight reduction were not further discussed in relation to existing research on similar interventions.
10.3390/nu16142331	Chueh et al.	COFIT application	Weight loss	Body weight	User Input	Body weight	Quasi-Experimental RWE Design	Observational	No	Prospective	Intergroup	n/a	Self-selected Comparator	Active Control limited app features	3a	10.297	56	Retrospective cohort study assessing an 8-week mobile app–based dietary intervention for weight loss in adults in Taiwan. Participants self-selected into either an intervention group, receiving structured dietary coaching from registered dietitians via a mobile app using the 2:1:1 portion plate and low-carbohydrate guidance, or a control group using the app for self-monitoring without coaching. Daily weight and dietary intake were logged via the app. Primary outcomes included percentage weight loss and BMI, while secondary outcomes included dietary adherence, record completeness, and macronutrient intake. Data were analyzed to evaluate differences between groups and identify behavioral predictors of weight loss.
10.2196/10422	Clement et al.	Kaia	Pain Reduction	Pain	User Input	Pain Level	Cohort Study	Observational	No	Retrospective	Intergroup	Cohorts bases on app version at the time of signup	No Direct Comparator	n/a	3b	1.251	168	The study uses user-reported pain and sleep data as well as app activity to compare effectiveness of two different app versions. Findings of reduction in pain in NRS was compared to clinical relevant effect as stated in literature. Increased app use resulted in high pain reduction which was descriptively discussed in relation to existing research of digital interventions on low back pain.

10.2196/36135	Cordella et al.	Constant Therapy	Speech, Language, Cognitive Rehabilitation	Domain scores (increase in difficulty level as percentage of total activities)	Device Generated	App Engagement	Cohort Study	Observational	No	Retrospective	Intergroup	Cohorts based on engagement	No Direct Comparator	n/a	3b	2.249	70	Retrospective study uses app engagement RWD to assess effectiveness of app to improve speech, language and cognitive skills. Outcomes were compared across users grouped by therapy frequency. Higher engagement was associated with greater improvements, dose-response trend was observed. Findings were descriptively discussed in relation to prior speech and language research on therapy intensity and dosage.
10.1097/PHM.0000000000002257	Delgado et al.	SimpleTherapy	Pain Reduction	Pain	User Input	Pain Level	Pre-Post Single-Group Study	Observational	No	Retrospective	Intra-Individual	n/a	No Direct Comparator	n/a	4	3.109	365	Study uses longitudinal RWD collected through in-app self-reports before exercise sessions to evaluate changes in musculoskeletal pain. Outcomes were compared across different engagement levels using mixed-effects models. Findings were discussed in relation to prior evidence on mhealth apps in lower back pain therapy.
10.2196/29583	Denis et al.	covidanosmia.eu web application	Symptom Improvement	Intensity of Olfactory Dysfunction	User Input	Olfactory ability	Pre-Post Single-Group Study	Observational	No	Prospective	Intra-Individual	n/a	No Direct Comparator	n/a	4	548	28	Prospective study uses self-reported RWD to assess app effectiveness on improving olfactory ability by calculating changes in olfactory function. Outcomes were compared within-person and between users who trained more/fewer days. Longer training duration was associated with higher rates of improvement. Findings were discussed in relation to RCT on olfactory training.
10.2196/52293	Dimeff et al.	Jaspr Health	Symptom Improvement	Agitation and Distress & Number of Adverse Events	User Input	Adverse Events (AEs)<br><br>Emotional State	Pre-Post Single-Group Study	Observational	No	Prospective	Intra-Individual	n/a	No Direct Comparator	n/a	4	962	335	Study analysis self-reported app data on agitation and distress collected during emergency department visit with a suicide prevention app. Pre–post measures of suicidal ideation and well-being were compared, with higher engagement linked to greater symptom improvement. Findings were descriptively contextualised to outcomes of a previously published RCT from this researcher group.
10.55563/clinexprheumatol/rng5n9	Dobies et al.	Sidekick Health RA programme	Symptom Improvement	Quality of Life	User Input	Symptom Severity	Cohort Study	Observational	No	Retrospective	Intergroup	Cohorts based on engagement	No Direct Comparator	n/a	3b	635	112	Retrospective study with self-reported rheumatoid arthritis related symptom RWD to evaluate effectiveness of app to improve symptoms. Symptom improvement were analysed within-person (pre-post change) and between groups based on app engagement. Higher engagement was associated with greater improvements.
10.2196/48435	Eguchi et al.	Calomama Plus	Weight loss	Body weight	User Input	Body weight	Pre-Post Single-Group Study	Observational	No	Retrospective	Inter-Individual	Subgroups based on engagement levels	No Direct Comparator	n/a	4	26.589	730	Study uses longitudinal RWD collected at user-selected timepoints to analyse change in body weight over study period. Higher engagement levels, measured by tracking frequency and duration of app use, were consistently associated with greater weight loss. Findings were descriptively discussed in relation to existing research on digital self-monitoring and weight loss in Asian populations.

10.1186/s12884-023-06004-7	Feng et al.	smartphone based weight management application (App)	Avoid Complication	Number of caesarean sections	System Generated	Caesarean section	Randomised controlled trial	Interventional	Yes	Prospective	Intergroup	n/a	Randomized Comparator	Usual Care (Clinical Data)	2	281	203	Randomized controlled trial evaluating the impact of a smartphone-based weight management app on cesarean section (CS) rates among overweight and obese pregnant women in China. Women were randomized to use the app alongside usual care or to receive usual care alone. The primary outcome, passively collected based on hospital clinical records, was CS rate. The intervention significantly reduced overall CS rate, with effects most pronounced in the overweight subgroup.
10.2196/24030	Fundoiano-Hershcovitz et al.	Dario digital therapeutics solution	Disease Management	Blood glucose level	Device Generated	Blood glucose level	Cohort Study	Observational	No	Retrospective	Intergroup	Cohorts based on engagement	No Direct Comparator	n/a	3b	998	365	Retrospective study uses longitudinal RWD on blood glucose readings and passively sensed app engagement to analyse effectiveness of app. Findings show correlation between higher app engagement and stronger reduction in blood glucose levels. Researchers showed that when an individual increased their tagging activity in one month, their blood glucose level tended to improve the next month - suggesting a quasi-causal relationship. Findings were descriptively discussed in relation to existing literature on digital behaviour interventions.
10.1089/dia.2022.0134	Grady et al.	OneTouch Reveal (OTR) mobile app	Disease Management	Blood glucose level	Device Generated	Blood glucose level	Pre-Post Single-Group Study	Observational	No	Retrospective	Intra-Individual	n/a	No Direct Comparator	n/a	4	17.777	90	Study uses longitudinal RWD of sensor blood glucose data and app engagement to assess apps effectiveness in controlling blood glucose. Findings were compared within-person at start and end of program and based on app engagement levels. More frequent and longer app use was associated with greater improvements in glucose levels.
10.1007/s13300-023-01415-3	Grady et al.	OneTouch Reveal (OTR) mobile app	Disease Management	Blood glucose level	Device Generated	Blood glucose level	Pre-Post Single-Group Study	Observational	No	Retrospective	Intra-Individual	n/a	No Direct Comparator	n/a	4	39.826	180	Retrospective single-group pre-post cohort study evaluating the impact of a Bluetooth®-connected blood glucose meter and companion mobile app on glycemic outcomes among over 55,000 people with type 1 or type 2 diabetes. Blood glucose data were passively and automatically collected via Bluetooth® from the meter to the app and analyzed retrospectively from baseline through 90- and 180-day timepoints.
10.1177/00048674231183641	Guiney et al.	Just a thought	Symptom Improvement	Psychological Distress	User Input	Symptom Severity	Pre-Post Single-Group Study	Observational	No	Retrospective	Intra-Individual	n/a	No Direct Comparator	n/a	4	14.844	540	Study uses RWD self-reported distress and program completion rate to analyse changes in mental distress. Findings were compared within-person and completion rate. Higher lesson completion was associated with greater reductions in distress, with the largest improvements occurring in the first half of the course. Findings were descriptively discussed in relation to prior RCT evidence on iCBT effectiveness.

10.2147/CEOR.S402357	Hadzi Boskovic et al.	Aripiprazole tablets with sensor	Adherence improvement	Psychiatric pharmacy claims & Inpatient and outpatient admissions & psychiatric ED claims	System Generated	ED Admissions<br><br>Medication use	Quasi-Experimental RWE Design	Observational	No	Retrospective	Intergroup	n/a	Propensity Score Matched Comparator	Usual Care (Claims Data)	3a	288	540	Quasi-experimental retrospective cohort study using real-world data from passively collected insurance claims data. Intergroup comparisons were conducted between aripiprazole with sensor (AS) users and propensity score–matched controls receiving standard oral antipsychotics (4:1 ratio). Data covered a 3-month baseline and 6-month follow-up period. Outcomes included treatment adherence and psychiatric healthcare resource utilization.
10.2196/58551	Hall et al.	Leva Pelvic Health System	Symptom Improvement	Urogenital Distress	User Input	Symptom Severity Questionnaire	Pre-Post Single-Group Study	Observational	No	Retrospective	Intra-Individual	n/a	No Direct Comparator	n/a	4	947	540	Study uses participant-reported symptom severity scores to calculate within-person changes pre- and post intervention to proof effectiveness of intervention to improve health outcome. Study findings of improvements in symptom severity were discussed in relation to previous research on same device.
10.1186/s13033-023-00592-9	Harty et al.	Space from Depression, Space from Anxiety, Space from Depression and Anxiety, and Space from Generalised Anxiety Disorder by [illegible]	Symptom Improvement	Depressive Symptoms & Anxiety Severity & Work and Social Adjustment	User Input	Symptom Severity Questionnaire	Pre-Post Single-Group Study	Observational	No	Retrospective	Intra-Individual	n/a	No Direct Comparator	n/a	4	3.236	365	Study uses longitudinal depression and anxiety questionnaire RWD to proof effectiveness of app to improve symptoms. Findings were compared within users based on app engagement. Greater app engagement was associated with stronger symptom improvements. Clinically meaningful changes were observed and descriptively discussed in relation to existing research on digital CBT and RCT benchmarks.
10.3389/fdgth.2023.1128553	Holmstrand et al.	Omron	Blood pressure management	Blood pressure	Device Generated	Blood pressure	Quasi-Experimental RWE Design	Interventional	No	Prospective	Intergroup	n/a	Propensity Score Matched Comparator	Usual Care (Blood pressure Data from EHR)	3a	204	180	Quasi-experimental matched cohort study using real-world data to assess the clinical effectiveness of a digital hypertension management platform. Home blood pressure readings were passively collected via connected sensors and used to monitor changes in BP over a 6-month period. Electronic health records (EHR) provided baseline clinical variables (e.g., BP, lipid levels, diabetes status). Intergroup comparisons between OMRON platform users and matched controls evaluated changes in BP control.
10.1002/osp4.757	Huntriss et al.	SIMPLE mobile application	Weight loss	Body weight	User Input	Body weight	Cohort Study	Observational	No	Retrospective	Intergroup	Cohorts based on engagement	No Direct Comparator	n/a	3b	36.950	365	Study uses longitudinal RWD on body weight collected at predefined timepoints to evaluate the apps effectiveness to support weight loss. Findings were compared across engagement levels, with higher in-app activity associated with greater weight loss. A clear dose-response relationship was observed, with the highest engagement group achieving the largest proportion of clinically significant weight loss. Findings were descriptively discussed in relation to existing evidence on intermittent fasting and app-based weight loss interventions.

10.2196/15189	Idris et al.	OurPath / Second Nature	Weight loss	Body weight	Device Generated	Body weight	Pre-Post Single-Group Study	Observational	No	Retrospective	Intra-Individual	n/a	No Direct Comparator	n/a	4	896	365	Retrospective single-group pre-post cohort study evaluating the effectiveness of the OurPath (Second Nature) digital lifestyle program for weight management among UK adults with obesity or type 2 diabetes. Participants were self-referred or NHS-referred and used a smartphone app combining education, coaching, and health tracking. Weight data were collected passively via wireless scales at baseline, 6 months, and 12 months, enabling repeated intra-individual comparisons.
10.2196/12106	Inkster et al.	Wysa App	Symptom Improvement	Depressive Symptoms	User Input	Depression severity	Pre-Post Single-Group Study	Observational	No	Prospective	Inter-Individual	Subgroups based on user characteristics and engagement	No Direct Comparator	n/a	4	123	55	Observational pre-post single-group study using actively collected real-world data via in-app self-report surveys. Depressive symptoms (PHQ-9) were collected at user-defined intervals. No control group was used. The study included inter-individual comparisons based on user characteristics and engagement to explore associations with symptom change.
10.1080/13625187.2019.1581164	Jennings et al.	Dynamic Optimal Timing (Dot) / Clue	Fertility management	Typical Use effectiveness & Perfect Use Effectiveness	User Input	Physiological parameters<br><br>Secual acitivity, Pregnancy and Mode of pregnancy planning	Cohort Study	Observational	No	Prospective	Intergroup	Cohorts based on app usage patterns and outcomes	No Direct Comparator	n/a	3b	718	630	Study uses prospective observational data from 718 users over 13 menstrual cycles to assess the effectiveness of the Dot fertility app for pregnancy prevention. Participants provided cycle dates, daily sexual activity, and pregnancy intention. Perfect- and typical-use failure rates were calculated using life-table analysis. Pregnancy outcomes were verified through self-reports and follow-up. The study found a typical-use failure rate of 5.8% and a perfect-use failure rate of 1.0%, indicating that Dot's effectiveness is comparable to other user-initiated contraceptive methods.
10.2196/18660	Kriventsov et al.	Diabits	Disease Management	Blood glucose level	Device Generated	Blood glucose level<br><br>Time in euglycemic range	Cohort Study	Observational	No	Retrospective	Intragroup	Cohorts based on engagement	No Direct Comparator	n/a	3b	280	730	Retrospective cohort study evaluating the accuracy of a smartphone-based glycemic prediction app (Diabits) and its association with blood glucose control under free-living conditions. Continuous glucose monitoring (CGM) data from over 500 users were analyzed to assess real-time prediction accuracy using clinical error grids. Additionally, blood glucose control metrics were compared across days with varying app usage frequency among 280 long-term users.
10.2196/47638	Kumbara et al.	The BlueStar (Welldoc)	Disease Management	Blood glucose level	Device Generated	Blood glucose level<br><br>Time in euglycemic range	Pre-Post Single-Group Study	Observational	No	Retrospective	Intra-Individual	n/a	No Direct Comparator	n/a	4	52	90	Retrospective, single-arm observational study evaluating the impact of a combined real-time continuous glucose monitoring (RT-CGM) and AI-powered digital health app (BlueStar) on glycemic outcomes and engagement in 52 adults with type 2 diabetes over 3 months. Intra-individual comparisons were made between baseline and follow-up glycemic metrics.

10.2196/45469	Lehmann et al.	Oviva	Weight loss	Body weight	User Input	Body weight	Cohort Study	Observational	No	Retrospective	Intergroup	Predefined cohorts based on app engagement	No Direct Comparator	n/a	3b	19.211	180	Retrospective observational cohort study using real-world data to evaluate the association between early app engagement and weight loss in a large-scale blended-care weight management program. Patients from Switzerland, Germany, and the United Kingdom were assigned to higher- or lower-engagement cohorts based on app usage within the first 3 months. Propensity score matching balanced cohorts on demographics, diagnosis, and coaching interactions. Multivariate linear regression models assessed percentage weight loss at 3 and 6 months, adjusting for baseline characteristics. Weight data were self-reported by patients via the mobile app and recorded as part of routine digital care.
10.1186/s12888-022-04411-2	Liang et al.	Good Sleep 365 Days	Symptom Improvement	Sleep Quality	User Input	Sleep Quality	Cohort Study	Observational	No	Retrospective	Intergroup	Cohorts based in diagnostic group	No Direct Comparator	n/a	3b	6.002	84	Retrospective observational cohort study evaluating the impact of digital cognitive behavioral therapy for insomnia (dCBT-I), alone or in combination with medication, on sleep quality among 6,002 adult patients with insomnia complaints in a psychiatric hospital sleep clinic. Participants were grouped by diagnosis (insomnia, anxiety, anxiety with insomnia, or depression) and by treatment type (dCBT-I monotherapy, medication alone, or combined therapy). Sleep quality was assessed using the Pittsburgh Sleep Quality Index (PSQI) at baseline, 8 weeks, and 12 weeks, allowing both intra-individual pre-post comparisons and intergroup comparisons across treatment modalities.
10.1038/s41746-024-01210-9	Liu et al.	CCT platform	Symptom Improvement	Cognitive Index	Device Generated	Cognitive ability<br><br>App Engagement	Cohort Study	Observational	No	Retrospective	Intergroup	Cohorts based on engagement	No Direct Comparator	n/a	3b	8.709	1.825	Retrospective study uses longitudinal task performance recordings and app engagement data to assess the apps effectiveness to improve cognitive function. Longterm follow up (5 years). Outcomes were compared across groups with varying training doses and frequencies. A dose–response relationship was observed. Findings were descriptively discussed in relation to prior meta-analyses and CCT efficacy trials. Large sample size (> 8700 participants).
10.1001/jamanetworkopen.2023.7597	Lu et al.	Good Sleep 365 Days	Symptom Improvement	Sleep Quality	User Input	Sleep Quality	Quasi-Experimental RWE Design	Observational	No	Retrospective	Intergroup	n/a	Propensity score–adjusted comparator	Three therapeutic modes (ie, dCBT-I, medication, and their combination)	3a	161	180	Retrospective cohort study evaluating the comparative effectiveness of digital cognitive behavioral therapy for insomnia (dCBT-I), medication therapy, and their combination, using data from a mobile app (Good Sleep 365). Adult patients with insomnia were assigned to treatment groups based on real-world prescriptions. Outcomes were compared across groups using inverse probability of treatment weighting (IPTW) to adjust for baseline confounders, forming a non-randomized, quasi-experimental comparator design. The primary outcome was change in sleep quality at 6 months, assessed via the Pittsburgh Sleep Quality Index (PSQI). Findings were descriptively discussed in relation to prior RCTs of dCBT-I.

10.2196/31353	Manning et al.	SWiPE	Symptom Improvement	Alcohol Consumption & Alcohol Craving	User Input	Alcohol Consumption<br><br>Alcohol Craving	Pre-Post Single-Group Study	Observational	No	Prospective	Intra-Individual	n/a	No Direct Comparator	n/a	4	1.309	30	Pre-post single-group study evaluating the feasibility, acceptability, and preliminary effectiveness of a personalized, gamified approach bias modification (ApBM) app (SWiPE) to reduce alcohol use in adults with hazardous drinking patterns. Participants (n=1309) completed baseline self-reports via online surveys and app-based measures of alcohol consumption, craving, and dependence. Alcohol use outcomes were self-reported weekly during training and again at 1-month follow-up, allowing intra-individual comparisons.
10.2196/10927	Mathiasen et al.	Internetpsykiatrien	Symptom Improvement	Depressive Symptoms & Anxiety Severity	User Input	Depression severity<br><br>Anxiety severity	Pre-Post Single-Group Study	Observational	No	Retrospective	Intra-Individual	n/a	No Direct Comparator	n/a	4	203	840	Pre-post single-group study evaluating the clinical effectiveness of guided internet-based cognitive behavioral therapy (iCBT) for adult depression and anxiety in routine secondary care. Participants (N=203) completed weekly self-reports of symptom severity (PHQ-9 for depression, GAD-7 for anxiety) via the treatment platform. Intra-individual comparisons were used to assess pre-post changes.
10.2196/47116	Mazéas et al.	Kiplin	Increase Physical Activity	Daily Step Count	Device Generated	Physical activity	Quasi-Experimental RWE Design	Observational	No	Retrospective	Intergroup	n/a	Self-selected Comparator	Usual Care (Registered for Platform but never used the app)	3a	4.819	1.095	Retrospective cohort study analyzing a gamified mobile health intervention to increase physical activity in adults. Over 4800 individuals who registered for a mobile app program were included, with participants either engaging in gamified step-counting challenges or serving as nonparticipating controls. Daily steps were tracked through smartphones or wearables. The intervention included team-based games with leaderboards, progress tracking, and motivational feedback. Multilevel models assessed within-individual step count changes from baseline through intervention and follow-up, with moderators such as age, initial activity level, and engagement. Step data were collected continuously and passively via linked devices in real-world conditions.
10.2196/26771	Mehta et al.	Youper	Symptom Improvement	Depressive Symptoms & Anxiety Severity	User Input	Anxiety severity<br><br>Depression severity	Pre-Post Single-Group Study	Observational	No	Retrospective	Intra-Individual	n/a	No Direct Comparator	n/a	4	4.517	30	Pre-post single-group study evaluating the preliminary effectiveness of a fully automated, AI-based mobile app (Youper) for anxiety and depression in a naturalistic setting. Participants (N=4517) completed self-reported measures of anxiety (GAD-7) and depression (modified PHQ-9) at baseline, 2 weeks, and 4 weeks. Intra-individual comparisons were used to assess pre-post changes.

10.2196/50330	Miller et al.	Balance weight gain prevention intervention	Weight loss	Body weight	System Generated	Body weight	Pragmatic Randomised controlled trial	Interventional	Yes	Prospective	Intergroup	n/a	Randomized Comparator	Usual Care (Clinical Data)	2	443	730	Pragmatic randomized controlled trial evaluating a digital weight gain prevention intervention (Balance) among adults with overweight or obesity receiving care at community health centers in North Carolina. Participants were randomized to either a 12-month digital program with tailored behavioral goals, coaching, and connected scale monitoring, or to receive healthy living materials and usual care. Outcomes were assessed using weight data extracted from routine electronic health records, with no study-specific follow-up visits. The study's pragmatic design integrated the intervention into real-world clinical workflows, used minimal exclusion criteria, and relied on existing care infrastructure.
10.2196/54754	Molander et al.	iCBT program	Symptom Improvement	Gambling Symptoms & Gambling Activity	User Input	Symptom Severity<br><br>Gambling Activity	Quasi-Experimental RWE Design	Observational	No	Retrospective	Intergroup	n/a	Propensity Score Matched Comparator	Usual Care (Registry Data)	3a	434	730	Quasi-experimental cohort study assessing a therapist-guided internet-delivered cognitive behavioral therapy (iCBT) program for gambling disorder in routine addiction care. Self-reported symptom severity was measured throughout treatment using the Gambling Symptom Assessment Scale (GSAS), collected at each treatment module. Effectiveness was further evaluated using registry-based outcomes on post-treatment healthcare engagement, including new psychiatric prescriptions, inpatient care episodes, and contact with social services. Intergroup comparisons were conducted between the iCBT group and a propensity score–matched sample receiving face-to-face psychological treatment in routine care, matched on age, gender, and psychiatric comorbidity.
10.2196/32351	Montgomery et al.	Happify, now Ensemble	Symptom Improvement	Well-being & Anxiety Severity	User Input	Symptom Severity<br><br>Well-being	Pre-Post Single-Group Study	Observational	No	Retrospective	Intra-Individual	n/a	No Direct Comparator	n/a	4	2.017	182	Pre-post single-group study analyzing real-world outcomes of a self-guided digital mental health intervention (Happify) in adults with self-reported cardiovascular disease risk factors (N=1803). Participants completed in-app assessments of anxiety (GAD-2) and subjective well-being over 6 weeks to 6 months. Mixed-effects models assessed intra-individual change and dose-response effects.
10.2196/44277	Nevoret et al.	Insulia	Disease Management	Blood glucose level	User Input	Blood glucose level	Cohort Study	Observational	No	Retrospective	Intergroup	Cohorts based on engagament	No Direct Comparator	n/a	3b	373	180	Retrospective analysis of real-world data from basal insulin–using patients with type 2 diabetes assessed the effectiveness of the Insulia app in supporting glycemic control. Participants were grouped by usage adherence. Pre-post within-person and between-group comparisons evaluated fasting glucose target attainment and HbA1c trends.

10.2196/16211	Parks et al.	Happify, now Ensemble	Symptom Improvement	Well-being	User Input	Well-being	Cohort Study	Observational	No	Retrospective	Intergroup	Cohorts based on condition	No Direct Comparator	n/a	3b	821	179	Retrospective observational study using real-world data from a publicly available digital platform to assess subjective well-being changes in users with and without chronic conditions. Participants (N=821) were grouped by condition status and usage intensity. Pre-post within-person changes and between-group comparisons were analyzed using mixed-effects models.
10.2147/COPD.S309372	Patel et al.	COPDPredict	Monitoring physiological parameters	Well-being & lung function	User Input, Device Generated	Well-being<br><br>Physiological parameters	Pre-Post Single-Group Study	Observational	No	Prospective	Intra-Individual	n/a	No Direct Comparator	n/a	4	90	180	Prospective single-group observational study evaluating the clinical utility of COPDPredict™, a digital remote monitoring system for predicting and managing COPD exacerbations. Adults with COPD (N=90) self-monitored daily symptoms via in-app wellbeing assessments, weekly spirometry, and periodic CRP testing. Real-time alerts were generated based on individualized baseline thresholds. Clinician-defined exacerbations were used to validate the algorithm's predictive performance.
10.1080/13625187.2020.1867844	Pearson et al.	Natural Cycles	Fertility management	Unintended pregnancies (Pearl Index)	User Input	Secual acitivity, Pregnancy and Mode of pregnancy planning	Pre-Post Single-Group Study	Observational	No	Prospective	Intra-Individual	n/a	No Direct Comparator	n/a	4	12.247	300	Pre-post single-group study evaluating the contraceptive effectiveness of a digital fertility-awareness based method (Natural Cycles) in routine real-world use. Participants (N=12,247) self-monitored fertility status daily via basal body temperature inputs through the app. Intra-individual data were used to assess typical- and perfect-use pregnancy rates over time.
10.21037/mhealth-23-66	Postel-Vinay et al.	Hy-Result	Blood pressure management	Blood pressure	User Input	Blood pressure	Cohort Study	Observational	No	Retrospective	Intergroup	Cohorts bases on recruitement	No Direct Comparator	n/a	3b	195	365	Retrospective, one-off collection of real-world data from home blood pressure monitoring via the Hy-Result web app. Reports were grouped by recruitment setting (primary care vs. hypertension center), and intergroup comparisons were made to assess compliance, BP control, and device usage.
10.2196/26401	Raffaelli et al.	M-sense	Symptom Improvement	Headache and migraine frequency & intensity	User Input	Headache information<br><br>Medication use	Pre-Post Single-Group Study	Observational	No	Retrospective	Intra-Individual	n/a	No Direct Comparator	n/a	4	1.545	210	Retrospective study uses longitudinal RWD from daily self-tracked headache diaries in app to analyse changes in symptoms. Within-person pre–post comparisons showed significant symptom reduction. Findings were descriptively discussed in relation to outcomes from behavioural headache interventions in the literature.
10.2196/52987	Richards et al.	OurPath / Second Nature	Weight loss	Body weight	Device Generated	Body weight	Pre-Post Single-Group Study	Observational	No	Retrospective	Intra-Individual	n/a	No Direct Comparator	n/a	4	1.130	730	Retrospective study uses longitudinal RWD collected through in-app self-assessments (PHQ-9, GAD-7) to evaluate mental health symptom changes in young adults engaging with a self-guided digital CBT intervention. Symptom changes were analysed both within individuals (pre–post) and between groups based on engagement level. Higher program completion was associated with greater symptom reduction. Findings were descriptively discussed in relation to existing digital mental health intervention studies.

10.1016/j.brat.2022.104084	Ritterband et al.	SHUTi (Sleep Health Using the Internet)	Symptom Improvement	Sleep Quality & Insomnia severity	User Input	Insomnia severity<br><br>Sleep Quality	Pre-Post Single-Group Study	Observational	No	Retrospective	Intra-Individual	n/a	No Direct Comparator	n/a	4	7.216	1.170	Retrospective study uses longitudinal sleep-related RWD to evaluate changes in insomnia symptoms. Within-person pre-post comparison showed symptom improvements after program completion. Findings were descriptively discussed in relation to outcomes from prior RCTs using the same intervention.
10.1016/j.jpsychores.2024.111864	Schläpfer et al.	CanRelax app 2.0	Symptom Improvement	Relaxation	User Input	Relaxation	Pre-Post Single-Group Study	Observational	No	Prospective	Intra-Individual	n/a	No Direct Comparator	n/a	4	91	70	The study uses longitudinal RWD collected through in-app pre-post ratings of perceived relaxation to evaluate the immediate effects of six relaxation techniques. Findings were compared across different techniques and within subject. Findings were descriptively discussed in relation to existing research on relaxation effectiveness.
10.2196/diabetes.8590	Stein et al.	Lark Weight Loss Health Coach AI (HCAI)	Weight loss	Body weight	Device Generated	Body weight	Pre-Post Single-Group Study	Observational	No	Retrospective	Intra-Individual	n/a	No Direct Comparator	n/a	4	70	105	Retrospective study uses self-reported weight and food intake RWD to evaluate apps capability to facilitate weight loss. Within-person comparison across levels of engagement to calculate weight change. Findings of weight reduction were discussed in relation to previous research on in-person lifestyle interventions.
10.2196/43775	Teepe et al.	Vivira	Pain Reduction	Pain & Physiological function	User Input	Pain Level<br><br>Motor function	Pre-Post Single-Group Study	Observational	No	Retrospective	Intra-Individual	n/a	No Direct Comparator	n/a	4	3.629	84	Retrospective study uses self-reported pain and physiological function RWD to assess change in symptoms over program use. Within-person comparison showed pain reduction and functional improvement during app use.
10.2196/38649	Teepe et al.	Vivira	Pain Reduction	Pain	User Input	Pain Level	Pre-Post Single-Group Study	Observational	No	Retrospective	Intra-Individual	n/a	No Direct Comparator	n/a	4	2.518	84	Retrospective study uses self-reported pain and functional RWD to assess change in symptoms over program use. Within-person (pre-post) comparison. Findings of pain reduction and functional improvement were descriptively discussed in relation to existing research.
10.3389/fpsyt.2024.1450615	Thorndike et al.	PDT; Somryst	Symptom Improvement	Insomnia Severity & Depressive Symptoms & Anxiety Severity	User Input	Insomnia severity<br><br>Symptom Severity Questionnaire	Pre-Post Single-Group Study	Observational	No	Prospective	Intra-Individual	n/a	No Direct Comparator	n/a	4	1.565	63	Prospective clinical study uses self-reported RWD on sleep and symptoms to assess effectiveness of app to improve symptoms. Findings were compared within-person and groups, with higher completion linked to greater symptom reduction. Long follow-up period (1 year). Findings were descriptively discussed in relation to prior RCTs and meta-analyses of CBT-I and digital sleep interventions.
10.2196/23227	Tu et al.	Health2Sync	Disease Management	Blood glucose level	User Input	Blood glucose level	Cohort Study	Observational	No	Retrospective	Intergroup	Cohorts based on engagement	No Direct Comparator	n/a	3b	2.036	365	Retrospective cohort study using real-world data to evaluate the effectiveness of a diabetes management app in routine clinical practice. HbA1c values were collected quarterly over 13 months from 2036 users and compared across usage-based groups (high, mid, low retention). Intra-individual and between-group comparisons assessed changes in glycemic control, revealing sustained HbA1c reductions correlated with continued app engagement.

10.3390/nu14102123	Ungersboeck et al.	Una health app	Disease Management	Blood glucose level	Device Generated	Blood glucose level	Pre-Post Single-Group Study	Observational	No	Prospective	Intra-Individual	n/a	No Direct Comparator	n/a	4	42	28	Prospective study uses RWD CGM and self-reported food intake data to evaluate blood glucose changes. Findings were analysed within-person. Improvements in iAUC and daytime AUC were observed, while mean glucose and time in hyperglycaemia remained unchanged. Findings were descriptively discussed in relation to earlier studies on CGM and personalised nutrition.
10.1186/s12891-022-05883-9	Vad et al.	Back Rx	Pain Reduction	Pain	User Input	Pain Level<br><br>Functional Disability	Pre-Post Single-Group Study	Observational	No	Prospective	Intra-Individual	n/a	No Direct Comparator	n/a	4	75	90	Prospective pilot study uses RWD pain level and function at baseline and end of program to evaluate the effectiveness of the app to reduce pain and improve function. Pre-post comparison of outcomes within person. Study descriptively discussed its results (on pain, function, adherence) in relation to prior RCTs, systematic reviews, and other digital health interventions for CLBP.
10.2196/25078	Venkatesan et al.	Vida health app	Symptom Improvement	Stress	User Input	Perceived Stress	Pre-Post Single-Group Study	Observational	No	Retrospective	Intra-Individual	n/a	No Direct Comparator	n/a	4	229	70	Retrospective study uses self-reported stress RWD to evaluate changes in perceived stress over program use. Findings were analysed within-person (pre-post) and on the basis of app engagement. Higher completion rate was associated with greater stress reduction. Results were descriptively discussed in relation to existing evidence on digital mindfulness interventions.
10.2196/21304	Venkatesan et al.	Vida health app	Symptom Improvement	Depressive Symptoms & Anxiety Severity	User Input	Depressive Symptoms<br><br>Anxiety severity	Pre-Post Single-Group Study	Observational	No	Retrospective	Intra-Individual	n/a	No Direct Comparator	n/a	4	323	84	Retrospective study uses self-reported depression and anxiety symptoms (PHQ8, GAD7) and app usage to evaluate symptom changes over program use. Findings symptom improvements were associated with higher app engagement. Findings were discussed in relation to in-person CBT trials and other digital CBT programs.
10.2196/38005	Venkatesan et al.	Vida health app	Symptom Improvement	Depressive Symptoms & Anxiety Severity	User Input	Depressive Symptoms	Pre-Post Single-Group Study	Observational	No	Retrospective	Intra-Individual	n/a	No Direct Comparator	n/a	4	1.512	84	Retrospective study uses self-reported depression and anxiety symptoms (PHQ8, GAD7) and app usage to evaluate symptom changes over program use. Within-person (pre-post) comparison and app engagement. Findings of reduced depression and anxiety symptoms during app use were discussed in relation to previously published RCT results on in-person and digital CBT effectiveness. Higher engagement resulted in greater symptom improvement.
10.2196/mhealth.5960	Wahle et al.	Mobile Sensing and Support (MOSS)	Symptom Improvement	Depressive Symptoms & Relation between adherence and change	User Input	Depressive Symptoms	Pre-Post Single-Group Study	Observational	No	Retrospective	Intra-Individual	n/a	No Direct Comparator	n/a	4	36	14	Retrospective study use self-reported depression RWD (PHQ-9) to assess change in depressive symptoms during program use. Findings of improved depressive symptoms and correlates with higher app engagement. No discussion of results in relation to existing literature.

10.2147/JPR.S412081	Wang et al.	Digital MSK Program	Pain Reduction	Opiod use	System Generated	Medication use	Quasi-Experimental RWE Design	Observational	No	Retrospective	Intergroup	n/a	Propensity Score Matched Comparator	Usual Care (Claims Data)	3a	8.390	365	Quasi-experimental cohort study evaluating the effectiveness of a digital musculoskeletal (MSK) program in reducing opioid initiation. Medical and pharmacy claims data were used to compare opioid prescriptions over 12 months between program participants and propensity score–matched physical therapy patients, with multivariate regression controlling for demographics, comorbidities, and baseline MSK-related healthcare use.
10.3390/cancers16051020	Wolff et al.	PINK! Coach	Disease Management	Physical activity & Body weight	User Input, Device Generated	Physical activity<br><br>Body weight	Pre-Post Single-Group Study	Observational	No	Retrospective	Intra-Individual	n/a	No Direct Comparator	n/a	4	776	90	Retrospective study uses body weight and physical activity data to evaluate BMI and physical activity changes. Comparisons were made within individuals over time and across subgroups (e.g., BMI categories, AHT/CHT therapy types). Results showed that BMI remained stable overall, with significant reductions in overweight patients under AHT and in those undergoing CHT. Physical activity (steps and active minutes) significantly increased. Findings were descriptively discussed in relation to existing literature on weight gain during cancer treatment.
10.2196/43489	Wu et al.	Omada for Hypertension	Blood pressure management	Blood pressure	Device Generated	Blood pressure<br><br>Body weight	Pre-Post Single-Group Study	Observational	No	Retrospective	Intra-Individual	n/a	No Direct Comparator	n/a	4	1.117	365	Retrospective study uses passively sensed blood pressure and body weight RWD and app engagement to analyse within subject pre-post changes in blood pressure. Findings of clinically relevant reduction in blood pressure were associated with higher app engagement. Clinical meaningful reduction was compared to recent meta-analysis on changes in blood pressure. Findings were discussed in relation to a previous study linking app engagement and blood pressure control.
10.1177/20552076231178616	YangToh et al.	Intellect	Symptom Improvement	Resilience & mood	User Input	Mood Level<br><br>Resilience Score	Pre-Post Single-Group Study	Observational	No	Retrospective	Intra-Individual	n/a	No Direct Comparator	n/a	4	515	30	Retrospective study uses self-reported mood symptom RWD to evaluate change in symptom severity. Within-person and across group comparison. Daily app engagement was associated with improved next-day mood and resilience.
10.2196/17455	Zhang et al.	Lilly Connected Care Program (LCCP)	Disease Management	Blood glucose level	Device Generated	Blood glucose level	Cohort Study	Observational	No	Retrospective	Intergroup	Cohorts based on engagement	No Direct Comparator	n/a	3b	5.011	84	Retrospective, one-off collection of RWD on passively sensed blood glucose and course completion was analysed to assess effectiveness of app to improve glycemic control. Participant grouping on basis of course completion. Pre-post within person and among group comparison. Findings of improved glycimic control and correlation with usage pattern were descriptively discussed in relation to existing research.
10.1089/dia.2024.0169	Zivkovic et al.	mySugr	Disease Management	Blood glucose level	Device Generated	Blood glucose level	Pre-Post Single-Group Study	Observational	No	Retrospective	Intra-inidividual & intragroup	n/a	No Direct Comparator	n/a	4	1.271	180	Retrospective RWD on blood glucose from user who transitioned from self-monitoring to CGM was analysed to measure effectiveness of app. Glycemic control outcomes were compared within-subjects before and after transitioning. Findings of improved health outcomes were descriptively discussed in relation to existing literature.

OSF-Protocol Deviations

In our original protocol (https://osf.io/3f5ur/), the primary research question was phrased in terms of whether mHealth applications routinely generate RWD that could support evidence of their effectiveness. While this reflected our conceptual intent, we realised during manuscript development that the phrasing could be misinterpreted as focusing on the apps themselves rather than on published studies evaluating them.

To improve clarity, we refined the wording to make explicit that our unit of analysis is peer-reviewed evaluation studies, and that our interest lies in how these studies use naturally emerging RWD to generate real-world evidence (RWE) about mHealth effectiveness.

This wording change clarifies our analytical focus but does not lead to any change in the study's scope, inclusion criteria, or methodology. Our objectives, data sources, and interpretation of findings remain fully aligned with the original protocol.

Original Wording Research Questions:

To what extent does the use of mHealth applications currently (routinely) generate real-world data that could be useful for (continuously) demonstrating their effectiveness in improving care?

1. Which types of patient-focused mHealth applications currently incorporate naturally emerging RWD for effectiveness evaluation?
2. What categories and types of naturally emerging RWD from routine app use are being collected in these applications?
3. Which study designs are used to assess the effectiveness of specific mHealth applications using naturally emerging RWD, and how do they align with regulatory and scientific recommendations?

Reworded Research Questions:

To what extent is real-world data (RWD) generated through routine use of mHealth applications used to (continuously) demonstrate their effectiveness in improving care, as reflected in peer-reviewed studies?

1. Which types of patient-focused mHealth applications currently incorporate naturally emerging RWD in studies for effectiveness evaluation?
2. What categories and types of naturally emerging RWD from routine app use are being reported in these studies?
3. Which study designs are used to assess the effectiveness of specific mHealth applications using naturally emerging RWD, and how do they align with regulatory and scientific recommendations?

References

1. Swift, B. *et al.* Innovation at the Intersection of Clinical Trials and Real-World Data Science to Advance Patient Care. *Clinical and Translational Science* vol. 11 450–460 Preprint at https://doi.org/10.1111/cts.12559 (2018).
2. U.S. Food & Drug Administration. *Framework for FDA's Real-World Evidence Program*. https://www.fda.gov/media/120060/download (2018).
3. OCEBM Table of Evidence Working Group *et al. Oxford Centre for Evidence-Based Medicine 2011 Levels of Evidence*. https://www.cebm.ox.ac.uk/resources/levels-of-evidence/ocebm-levels-of-evidence (2011).
4. Frieden, T. R. Evidence for Health Decision Making — Beyond Randomized, Controlled Trials. *New England Journal of Medicine* **377**, 465–475 (2017).
5. Wang, M. *et al.* Single-arm clinical trials: design, ethics, principles. *BMJ Supportive and Palliative Care* Preprint at https://doi.org/10.1136/spcare-2024-004984 (2024).
6. Miroshnychenko, A. *et al.* Cohort studies investigating the effects of exposures: key principles that impact the credibility of the results. *Eye (Basingstoke)* vol. 36 905–906 Preprint at https://doi.org/10.1038/s41433-021-01897-0 (2022).
7. Linden, A. & Arbor, A. *Conducting Interrupted Time-Series Analysis for Single-and Multiple-Group Comparisons*. *The Stata Journal* vol. 15 (2015).
8. Guo, C. *et al.* Challenges for the evaluation of digital health solutions—A call for innovative evidence generation approaches. *npj Digital Medicine* vol. 3 Preprint at https://doi.org/10.1038/s41746-020-00314-2 (2020).

9. Tricco, A. C. *et al.* PRISMA Extension for Scoping Reviews (PRISMA-ScR): Checklist and Explanation. *Ann Intern Med* **169**, 467–473 (2018).
